# Amide-Driven Secondary Building Unit Structural Transformations between Zn(II) Coordination Polymers

**DOI:** 10.1021/acs.cgd.2c00520

**Published:** 2022-07-13

**Authors:** Daniel Ejarque, Teresa Calvet, Mercè Font-Bardia, Josefina Pons

**Affiliations:** † Departament de Química, Universitat Autònoma de Barcelona, Bellaterra, 08193 Barcelona, Spain; ‡ Departament de Mineralogia, Petrologia i Geologia Aplicada, Universitat de Barcelona, Martí i Franquès s/n, 08028 Barcelona, Spain; § Unitat de Difracció de Raig-X, Centres Científics i Tecnològics de la Universitat de Barcelona (CCiTUB), Universitat de Barcelona, Solé i Sabarís, 1-3, 08028 Barcelona, Spain

## Abstract

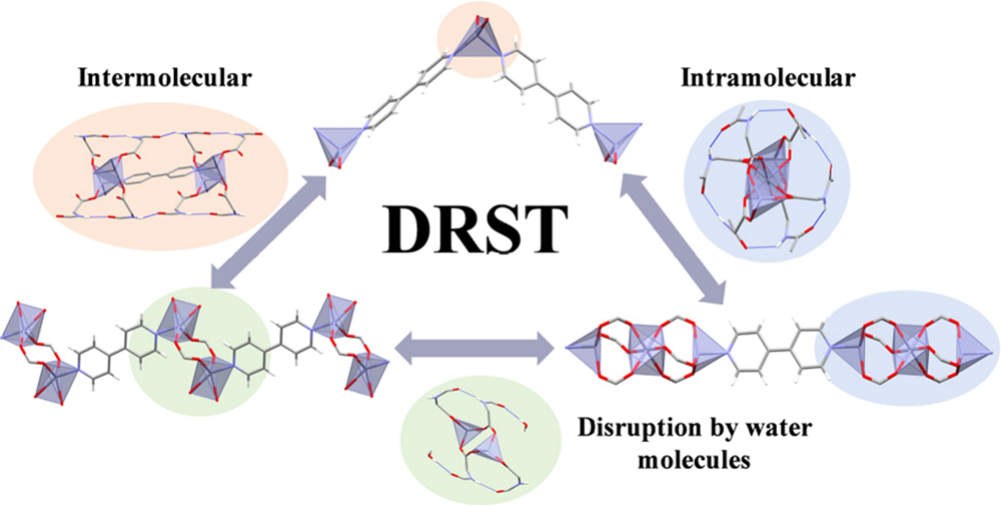

The behavior of coordination polymers (CPs) against external stimuli has witnessed remarkable attention, especially when the resulting CPs present reversible molecular arrays. Accordingly, CPs with these characteristics can lead to differences in their properties owing to these structural differences, being promising for their use as potential molecular switches with diverse applications. Herein, we have synthesized four Zn(II) CPs bearing α-acetamidocinnamic acid (HACA) and 4,4′-bipyridine (4,4′-bipy). The reaction between Zn(OAc)_2_·2H_2_O, HACA, and 4,4′-bipy yields {[Zn(ACA)_2_(4,4′-bipy)]·EtOH}_*n*_ (**1**), which was used for the formation of three CPs through dissolution–recrystallization structural transformations (DRSTs): {[Zn(ACA)_2_(4,4′-bipy)]·2MeOH}_*n*_ (**2**), {[Zn_2_(μ-ACA)_2_(ACA)_2_(4,4′-bipy)]·2H_2_O}_*n*_ (**3**), and {[Zn_3_(μ-ACA)_6_(4,4′-bipy)]·0.75CHCl_3_}_*n*_ (**4**). The study of the four crystal structures revealed that their secondary building units (SBUs) comprise monomeric, dimeric, and trimeric arrangements linked by 4,4′-bipy ligands. The fundamental role of the utilized solvent and/or temperature, as well as their effect on the orientation of the amide moieties driving the formation of the different SBUs is discussed. Furthermore, the reversibility and interconversion between the four CPs have been assayed. Finally, their solid-state photoluminescence has evinced that the effect of the amide moieties not only predetermine a different SBU but also lead to a different emission in **4** compared with **1**–**3**.

## Abstract 2

A CP with formula {[Zn(ACA)_2_(4,4′-bipy)]·EtOH}_n_ has been synthesized and used as a DRST precursor for the obtention of three CPs with variable SBUs, whose formation is dependent on the solvent and/or temperature. Solid-state photoluminescence measurements show the effect of the different SBUs leading to different emissions.

## Introduction

During the last decades, crystal engineering has arisen as an interdisciplinary field based on the rationalization of crystalline structures from their assemblies.^1^ Over time, the field has been branched into multicomponent solids (cocrystals, salts, and solvates)^2−4^ and coordination polymers (CPs).^5−7^ In this regard, CPs have attracted enormous interest not only for their structural diversity^8−10^ but also for their potential applications in gas sorption,^11^ sensing,^12^ or catalysis,^13^ among others.^14−16^ Their rational design consisting of the judicious selection of metal nodes and organic ligands has led to the formation of the desired secondary building units (SBUs), which are directly related with the properties of the resulting compounds.^17−19^

In this context, small changes on the synthetic conditions *inter alia* starting metal salt^20^ or precursor,^21^ metal–ligand ratio,^22^ temperature,^23^ or solvent^24^ can lead to the obtention of different CPs. In addition, their possible structural transformations triggered by external stimuli (e.g., solvent,^25^ light,^26^ heat,^27^ or synergic effects^28,29^) have attracted remarkable attention, paving the way for the obtention of new structures inaccessible through other synthetic methods.^30−32^ Besides, when these transformations show a reversible behavior, the CPs display promising applications in molecular capture,^33^ switches,^34^ and so on.^35^ Typically, these transformations are sorted into two groups, namely, solid-state structural transformations (SSSTs)^36^ and dissolution–recrystallization structural transformations (DRSTs).^37^ Both types lead to the thermodynamic product in specific synthetic conditions,^38^ albeit they drive to the formation of CPs with structural differences. The SSSTs occur in the solid state, which retain the main structural integrity of the initial compound. Differently, in DRSTs, the solvent plays the critical role of promoting the dissolution and cleavage of the coordination bonds of the initial compound followed by their rearrangement into a new complex.^39,40^ Therefore, SSSTs typically cause slight structural changes, while DRSTs involve more important transformations.^41^

The coordination, supramolecular interactions, and/or polarity of the solvent molecules usually lead to DRSTs.^42−44^ These differences generally result in changes in the coordination number,^45^ geometry,^40^ or dimensionality^46^ of the resulting CPs. Nonetheless, structural transformations of the SBUs are relatively scarce,^32,47−49^ and to the best of our knowledge, no examples of CPs presenting multiple alterations of its initial SBU by DRSTs can be found in the literature.

**Scheme 1:**
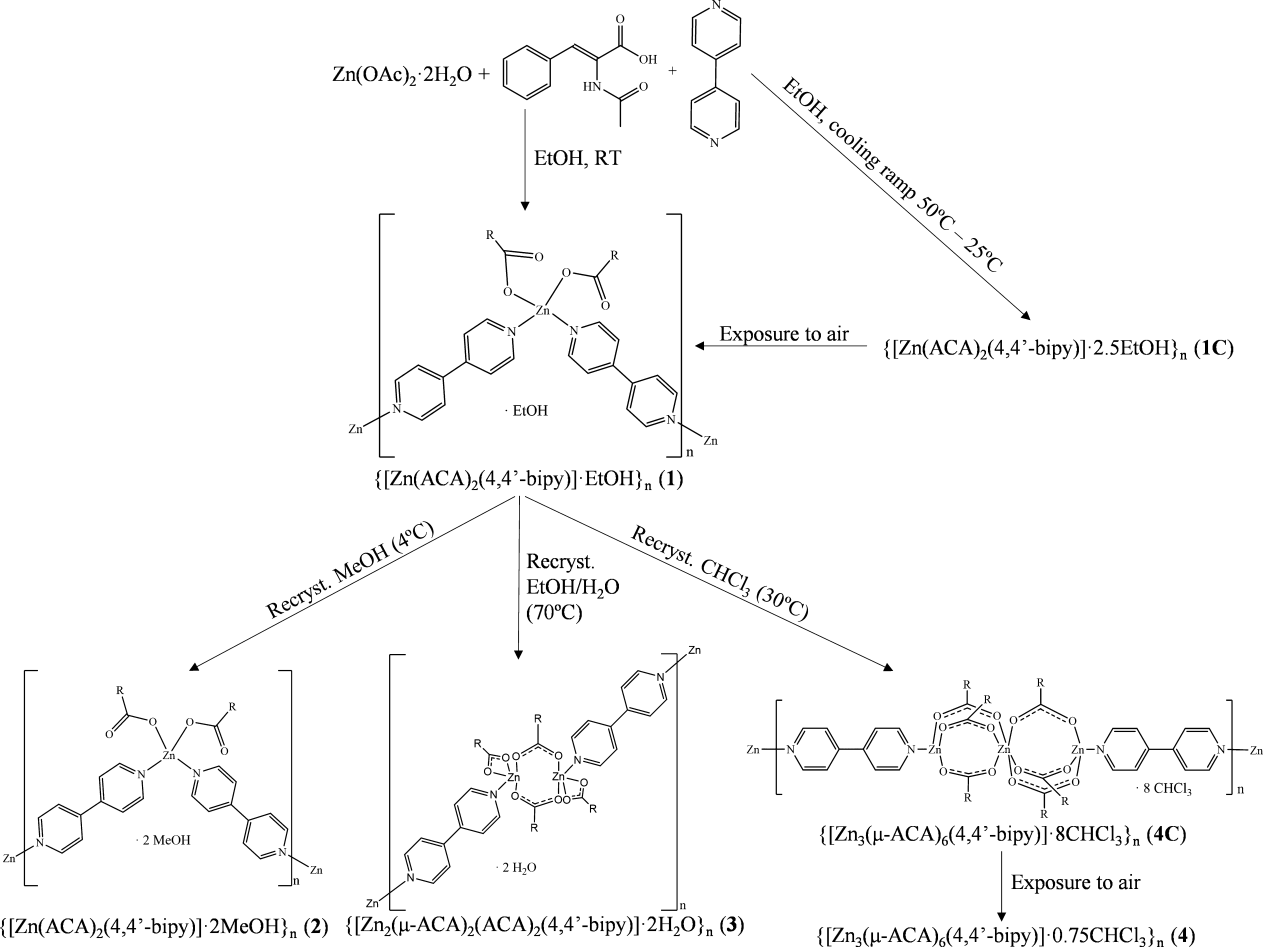
Outline of the Formation of Compounds **1**–**4**

In this scenario, our group has been working during the last years on the synthesis of CPs based on 4,4′-bipyridine^50^ and the preparation of Zn(II) and Cd(II) complexes bearing α-acetamidocinnamic acid (HACA) and different pyridine derivatives.^51−54^ These studies resulted in the obtention of a series of ACA-based compounds with different nuclearities encompassing monomeric, dimeric, trimeric, and even polymeric compounds extended through the ACA ligand. From them, the obtention of trimeric compounds with pinwheel arrays has especially attracted our attention, studying the effect of the intramolecular N–H···O_C=O_ synthon between vicinal ACA ligands, which plays an important role in the stabilization of the SBU.^52^ This effect led us to study the influence of the orientation of the acetamide moieties on their nuclearity, observing that they tend to form self-complementary N–H···O interactions that fold inward or outward the complex depending on the solvent. While in solvents with higher H-acceptor propensity such as MeOH or DMF, the acetamide moieties fold outward, displaying N–H···O_solvent_ interactions, in other solvents with less propensity to partake in H-bonding such CH_3_CN, the acetamide moieties orientates inward toward the formation of N–H···O_C=O_ intramolecular interactions.^55^ In addition, when EtOH was used, different orientations of the acetamide moieties were achieved, and the self-complementary interactions were not always obtained, observing competition with the uncoordinated oxygen atom from the carboxylate groups and solvent molecules.^51−54^ Therefore, aiming to extend our knowledge on ACA-based complexes, we have synthesized one CP from the reaction between Zn(OAc)_2_·2H_2_O, HACA, and 4,4′-bipy, namely, {[Zn(ACA)_2_(4,4′-bipy)]·EtOH}_*n*_ (**1**), which has been used as a precursor for DRSTs in different solvents (MeOH, EtOH/H_2_O, and CHCl_3_), yielding three CPs based on variable SBUs, namely, {[Zn(ACA)_2_(4,4′-bipy)]·2MeOH}_*n*_ (**2**), {[Zn_2_(μ-ACA)_2_(ACA)_2_(4,4′-bipy)]·2H_2_O}_*n*_ (**3**), and {[Zn_3_(μ-ACA)_6_(4,4′-bipy)]·0.75CHCl_3_}_*n*_ (**4**), respectively. Furthermore, the crystal structures of the four compounds have been elucidated and studied. For **1** and **4**, they correspond to {[Zn(ACA)_2_(4,4′-bipy)]·2.5EtOH}_*n*_ (**1C**) and {[Zn_3_(μ-ACA)_6_(4,4′-bipy)]·8CHCl_3_}_*n*_ (**4C**) (Scheme 1). The effects of the solvent in the orientation of the amide moieties driving the formation of different SBUs have been studied as well as the interconversion between these CPs. Finally, we further analyzed the solid-state photoluminescence of the resulting compounds.

## Experimental Section

### Synthesis of {[Zn(ACA)_2_(4,4′-bipy)]·EtOH}_*n*_ (**1**)

An EtOH solution (15 mL) of Zn(OAc)_2_·2H_2_O (100 mg, 0.456 mmol) was slowly added to an EtOH solution (15 mL) of HACA (187 mg, 0.911 mmol) and 4,4′-bipy (285 mg, 1.82 mmol) at RT. The resulting solution was stirred for 24 h until a white solid precipitated, and then, it was kept at −30 °C for 1 h. Then, the solid was filtered, washed twice with 10 mL of cold Et_2_O, and dried under vacuum. During the attempts to obtain single crystals, suitable colorless crystals for X-ray diffraction of {[Zn(ACA)_2_(4,4′-bipy)]·2.5EtOH}_*n*_ (**1C**) were obtained. These crystals were obtained using the same molar ratio as for **1**, but starting from 4.0 mg of Zn(OAc)_2_·2H_2_O and using a cooling ramp from 50 to 25 °C (SI: Figure S1). When these crystals were exposed to air, the loss of EtOH molecules yielded **1**, which was characterized (details are given in the Supporting Information).

### Obtention of **2**–**4**

These compounds were obtained by DRSTs of **1** (20 mg, 0.030 mmol) in MeOH, EtOH/H_2_O, and CHCl_3_, yielding different polymeric arrays with variable SBUs. DRST of **1** in MeOH results in the formation of {[Zn(ACA)_2_(4,4′-bipy)]·2MeOH}_*n*_ (**2**) after keeping the resulting solution in the fridge at 4 °C overnight, while DRST of **1** in EtOH/H_2_O (18/1) at 70 °C during 12 h at autogenous pressure affords {[Zn_2_(μ-ACA)_2_(ACA)_2_(4,4′-bipy)]·2H_2_O}_*n*_ (**3**). Finally, when DRST of **1** was conducted in CHCl_3_ at 30 °C during 12 h under autogenous pressure, single crystals of {[Zn_3_(μ-ACA)_6_(4,4′-bipy)]·8CHCl_3_}_*n*_ (**4C**) were obtained. The crystals of **2**, **3**, and **4C** were collected by filtration and dried under vacuum, obtaining {[Zn_3_(μ-ACA)_6_(4,4′-bipy)]·0.75CHCl_3_}_*n*_ (**4**) from compound **4C**, which unavoidably lost CHCl_3_ molecules upon air exposure. Therefore, the dried products **2**–**4** were characterized (see the Supporting Information).

### X-ray Crystallographic Data

For compounds **1C**, **2**, **3**, and **4C**, colorless prism-like specimens were used for the X-ray crystallographic analysis. For all the compounds, the frames were integrated using the Bruker SAINT software package using a narrow-frame algorithm. All hydrogen atoms were refined using a riding model (AFIX) with an isotropic temperature factor equal to 1.2, the equivalent temperature factor of the atom to which they are linked, and thus, the bond lengths of X–H were fixed. Crystal data and additional details of structure refinement for compounds **1C**, **2**, **3**, and **4C** are reported in Table 1 and in the Supporting Information. Complete information about the crystal structure and molecular geometry is available in the CIF format *via* CCDC 2168566 (**1C**), 2168567 (**2**), 2168568 (**3**), and 2168569 (**4C**).

**Table 1:** Crystal Data and Structure Refinement for **1C**, **2**, **3**, and **4C**

	**1C**	**2**	**3**	**4C**
empirical formula	C_74_H_86_N_8_O_17_Zn_2_	C_34_H_36_N_4_O_8_Zn	C_27_H_26_N_3_O_7_Zn	C_84_H_76_Cl_24_N_8_O_18_Zn_3_
formula weight	1490.24	694.04	569.88	2532.43
*T* (K)	100(2)	100(2)	100(2)	100(2)
wavelength (Å)	0.71073	0.71073	0.71073	0.71073
system, space group	triclinic, P1̅	monoclinic, C2/c	triclinic, P1̅	triclinic, P1̅
unit cell dimensions				
*a* (Å)	9.3672(9)	29.244(2)	9.0797(10)	13.4193(13)
*b* (Å)	13.9712(15)	5.7440(4)	9.4746(10)	14.4425(12)
*c* (Å)	16.0805(18)	19.4053(17)	15.4667(17)	16.0580(16)
α (°)	64.504(4)	90	78.255(4)	72.773(4)
β (°)	79.901(4)	92.191(3)	85.712(4)	66.418(4)
γ (°)	79.650(4)	90	87.664(4)	87.280(4)
*V* (Å^3^)	1857.2(3)	3257.3(5)	1298.6(2)	2715.3(4)
*Z*	1	4	2	1
*D*_calc_ (mg/m^3^)	1.332	1.415	1.457	1.549
μ (mm^–1^)	0.718	0.812	0.997	1.311
*F* (000)	782	1448	590	1278
crystal size (mm^–3^)	0.120 × 0.08 × 0.060	0.180 × 0.080 × 0.040	0.250 × 0.200 × 0.100	0.030 × 0.020 × 0.010
hkl ranges	–13 ≤ h ≤ 13, –17 ≤ k ≤ 20, 0 ≤ l ≤ 23	–41 ≤ h ≤ 41, –8 ≤ k ≤ 8, –24 ≤ l ≤ 27	–12 ≤ h ≤ 12, –13 ≤ k ≤ 13, –22 ≤ l ≤ 22	–16 ≤ h ≤ 16, –18 ≤ k ≤ 18, –20 ≤ l ≤ 20
θ range (°)	2.224–31.378	2.100–30.605	2.196–30.590	2.051–26.493
reflections collected/unique/[*R*_int_]	11,749/11749/0.0587	25,688/5003/0.1150	52,017/7846/0.0272	70,704/11166/0.1653
completeness to θ (%)	99.9	99.9	97.7	99.9
absorption correction	semi-empirical from equivalents	semi-empirical from equivalents	semi-empirical from equivalents	semi-empirical from equivalents
max. and min. transmission	0.7461 and 0.6903	0.7461 and 0.6646	0.7461 and 0.6688	0.7454 and 0.6774
refinement method	full-matrix least-squares on |*F*|^2^	full-matrix least-squares on |*F*|^2^	full-matrix least-squares on |*F*|^2^	full-matrix least-squares on |*F*|^2^
data/restrains/parameters	11,749/27/461	5003/0/216	7846/5/351	11,166/0/630
goodness-on-fit on F^2^	1.078	1.081	1.022	1.022
final *R* indices [*I* > 2σ(*I*)]	*R*_1_ = 0.0403, w*R*_2_ = 0.0931	*R*_1_ = 0.0570, w*R*_2_ = 0.1011	*R*_1_ = 0.0347, w*R*_2_ = 0.0895	*R*_1_ = 0.0734, w*R*_2_ = 0.1245
*R* indices (all data)	*R*_1_ = 0.0464, w*R*_2_ = 0.0998	*R*_1_ = 0.1300, w*R*_2_ = 0.1363	*R*_1_ = 0.0367, w*R*_2_ = 0.0915	*R*_1_ = 0.1262, w*R*_2_ = 0.1469
extinction coefficient	n/a	0.00112(15)	n/a	n/a
largest diff-peak and hole (e. Å^–3^)	1.455 and −0.823	0.818 and −1.013	1.340 and 0.846	1.380 and −1.214

## Results and Discussion

### Formation of **1**–**4**

Compound **1** was obtained by the addition of Zn(OAc)_2_·2H_2_O, HACA, and 4,4′-bipy in a 1:2:4 molar ratio, respectively, in EtOH as the solvent at RT. During the attempts to obtain single crystals suitable for X-ray diffraction, compound **1C** was obtained (details are provided in the Supporting Information).

The recrystallization of **1** in different solvents resulted in the formation of CPs consisting of variable SBUs linked by 4,4′-bipy ligands. Its DRST in MeOH afforded **2** based on monomeric SBUs. When **1** was dissolved in EtOH/H_2_O (18/1) at 70 °C, compound **3** consisting of dimeric SBUs was obtained, while the dissolution of **1** in CHCl_3_ at 30 °C resulted in the formation of **4C** based on trinuclear pinwheel arrays, which unavoidably lose CHCl_3_ molecules upon exposure to air, forming **4**. Because **2** and **4** are obtained through the direct DRSTs of **1** in MeOH and CHCl_3_, the solvent plays a crucial role in their transformations. Otherwise, the formation of **3** also need to achieve a temperature of 70 °C for conducting the DRST of **1** in the specified EtOH/H_2_O ratios and therefore, the synergistic effects of temperature and solvents are the driving forces that lead the structural transformation. In addition, the reaction of **1** in the absence of the solvent until temperatures of 90 °C or by mechanochemical grinding was also performed to ascertain that no SSSTs occur, obtaining the initial compound. Alternatively, compound **2** has also been obtained through the direct reaction between Zn(OAc)_2_·2H_2_O, HACA, and 4,4′-bipy in a 1:2:4 molar ratio in MeOH, while all the attempts to obtain **3** and **4** directly from the starting reagents were not successful.

### General Characterization

Compounds **1**–**4** were characterized by powder X-ray diffraction (PXRD), HR-ESI-MS, elemental analysis (EA), FTIR-ATR, ^1^H, ^13^C{^1^H}, and DEPT-135 NMR spectroscopies, and single crystal X-ray diffraction method (**1C**, **2**, **3** and **4C**). Furthermore, the thermal stability of **1** and **4** was studied *via* TG-DTA determinations. Phase purity of the bulk samples of **1**–**4** was verified by PXRD. Compounds **2** and **3** are stable under air exposure, while **1** and **4** lose solvent molecules upon exposure to air, resulting in a different packing of the crystal structures (**1C** and **4C**) compared with the powder (**1** and **4**) (SI: Figures S2–S5).^56^ EA of **2** and **3** agree with the proposed formula, while in **1C** and **4C**, a loss of solvent that is consistent with the PXRD data is observed. Considering the differences between **1/1C** and **4**/**4C** in the PXRD and EA, TG-DTA determinations of the solid samples of **1** and **4** were performed to verify the loss of solvent molecules and stability of these CPs. For **1**, a loss of one EtOH molecule (exp. 4.88%; calc. 6.81%) between 40 and 202 °C was observed, followed by its decomposition at 232 °C (SI: Figure S6). For **4**, any mass loss was observed until its decomposition, being stable until 244 °C (SI: Figure S7). Therefore, the TG-DTA measurements agree with the PXRD and EA results. The positive ionization mass spectra (ESI^+^-MS) of all the compounds were recorded using MeOH as the solvent. In these conditions, all the CPs show the peaks of the free ligands at m/z 157.0762 (100%) [4,4′-bipy + H]^+^ and 228.0622 (100%) [HACA + Na]^+^ as well as Zn fragments with monomeric, dimeric, and trimeric patterns at m/z 473.0697 (100%) [Zn(ACA)_2_ + H]^+^, 495.0492 (100%) [Zn(ACA)_2_ + Na]^+^, 740.0591 (79%) [Zn_2_(ACA)_3_]^+^, 967.1126 (74%) [Zn_2_(ACA)_4_ + Na]^+^, and 504.0268 (40%) [Zn_3_(ACA)_4_]^2+^ (SI: Figure S8).

In the FTIR-ATR spectra, the absence of bands between 2704–2405 cm^–1^ corresponding to ν(O–H)_HACA_, combined with a strong peak at 1637 cm^–1^ attributable to ν(COOH)_HACA_, indicates that the HACA is deprotonated in the four CPs. The spectra display the typical bands between 1608–1523 cm^–1^ for ν_as_(COO) and 1410–1372 cm^–1^ for ν_s_(COO). The difference between these bands [Δ = ν_as_(COO) - ν_s_(COO)] is 236 cm^–1^ (**1**), 207 cm^–1^ (**2**), 175 and 113 cm^–1^ (**3**), and 184 cm^–1^ (**4**), suggesting monodentate (**1** and **2**), bidentate chelate (**3**), and bidentate bridged (**3** and **4**) coordination modes of the carboxylate groups.^57,58^ Moreover, additional groups of the ACA ligand such as the NH and C=O groups have also been identified in all of the spectra, as well as the ν(C–H)_ar_, ν(C=C/C=N), δ_ip_(C–H), and δ_oop_(C–H) bands from the aromatic rings.^59^ The presence of solvent molecules allows further identification of some specific bands in **1**–**3**, observing different peaks at 3455–3378 cm^–1^ (**1**), 3646 cm^–1^ (**2**), and 3581 and 3496 cm^–1^ (**3**) attributable to ν(O–H) (SI: Figures S9–S12).

The ^1^H NMR spectra of **1**–**4** were recorded in DMSO-*d*_6_ solutions to distinguish between the different molar ratios of the ligands (ACA:4,4′-bipy) (SI: Figures S13–S16). The spectra of the four complexes show a signal between 9.20 and 9.17 ppm attributable to the amide proton atom of ACA, while the signals of the aromatic protons of 4,4′-bipy and ACA appear between 8.73–7.83 and 7.50–7.28 ppm, respectively. Moreover, a signal at 7.24–7.23 ppm corresponding to the alkene proton is also observed. Finally, the methyl protons appeared at 1.96–1.95 ppm. The ^1^H NMR spectra of **1**–**4** confirm the different ACA:4,4′-bipy ratios of the CPs, being 2:1 (**1** and **2**), 4:1 (**3**), and 6:1 (**4**), which is in line with the data from the X-ray crystallographic analysis.^59^

The ^13^C{^1^H} and DEPT-135 NMR spectra of **1**–**4** have also been recorded in DMSO-*d*_6_ solutions (SI: Figures S17–S20). The spectra of the four complexes display the band assignable to the carbon atom of the carbonyl group at 170.6–170.3 ppm, while the carbon atom from the carboxylate group appears between 168.7–168.4 ppm. The carbon atoms from 4,4′-bipy are also observed between 150.7–121.4 ppm. In this region, the two carbon atoms from the double bond of ACA are also observed at 135.2–135.1 and 128.4–128.2 ppm. Additionally, the aromatic carbon atoms from ACA are located between 129.8 and 128.3 ppm. Finally, the methyl carbon atoms from ACA are found to be at 23.1–23.0 ppm.

### Structural Description of {[Zn(ACA)_2_(4,4′-bipy)]·2.5EtOH}_*n*_ (1C) and {[Zn(ACA)_2_(4,4′-bipy)]·2MeOH}_*n*_ (**2**)

**Figure 1:**
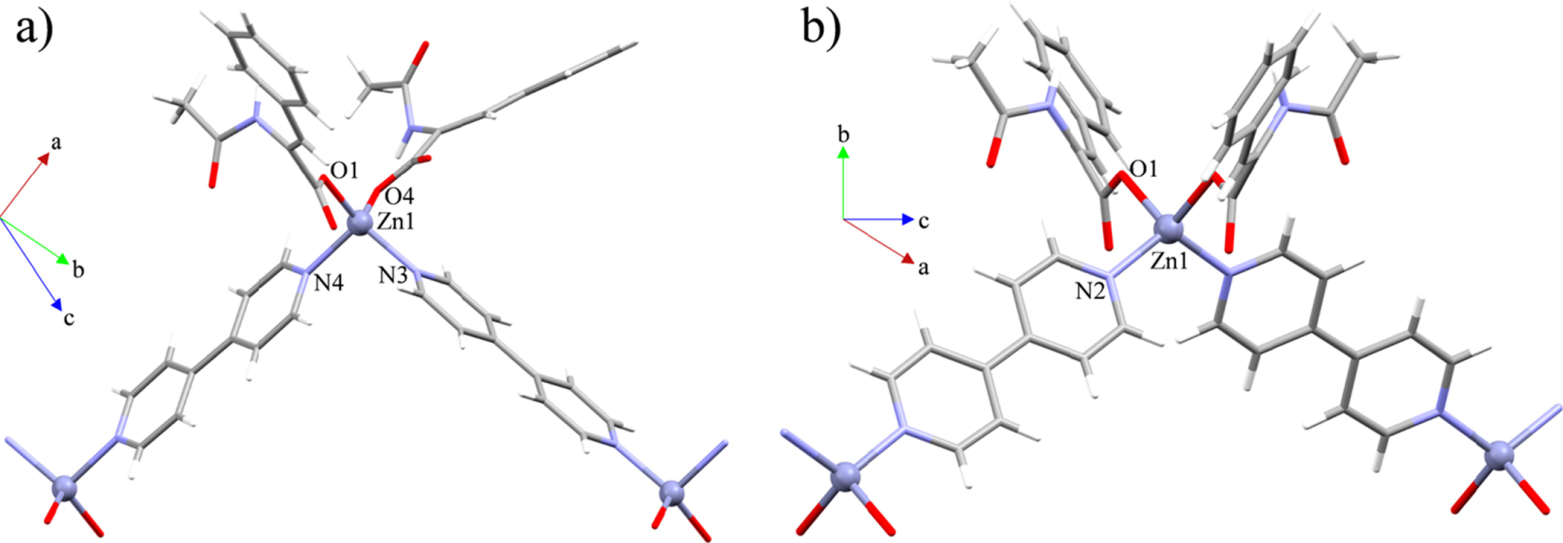
Molecular structure of compounds (a) **1C** and (b) **2**.

Compounds **1C** and **2** belong to the triclinic P1̅ and monoclinic C2/c space groups, respectively. They consist of 1D *zig-zag* CPs expanded along the [101̅] (**1C**) and [001] (**2**) directions. These polymers are assembled by monomeric SBUs with a [ZnO_2_N_2_] (**1C** and **2**) *core* composed by two monodentate (μ_1_-η^1^) ACA ligands as well as one 4,4′-bipy, which joins together the monomeric SBUs (Figure 1). The evaluation of the geometry has been done using the low continuous shape measure (CShM) value *S*.^60^ Both S values fit with a tetrahedral geometry (**1C**: 0.946; **2**: 0.630) (SI: Table S1), which consistently agree with the τ_4_ values (**1C**: 0.84; **2**: 0.91).^61^ The unit cell of **1C** contains two equivalent polymeric chains and five EtOH molecules. These solvent molecules are in voids generated by the supramolecular scaffold with an accessible volume of 181.11 Å^3^ (9.8% of the unit cell volume). For **2**, the supramolecular scaffold generates voids with an accessible volume of 30.78 Å^3^ (0.9% of the unit cell volume),^62^ which contain two MeOH molecules. The bond lengths and bond angles for both CPs oscillate between 1.9211(11)-2.0666 (13) Å and 97.94(5)-129.27 (5)° (**1C**), and 1.965(2)-2.022 (2) Å and 100.30(13)-116.89 (14)° (**2**), presenting similar values than other Zn(II) four-coordinated CPs based on monomeric SBUs linked by 4,4′-bipy spacers (Tables 2 and 3).^63−65^

**Table 2:** Selected Bond Lengths (Å), Bond Angles (°), and Intermolecular Interactions (Å) for **1C**

bond lengths (Å)				
Zn(1)-O(1)		1.9211(11)	Zn(1)-N(3)	2.0268(13)
Zn(1)-O(4)		2.0093(11)	Zn(1)-N(4)	2.0666(13)
bond angles (°)				
O(1)-Zn(1)-O(4)		111.79(5)	O(4)-Zn(1)-N(3)	107.52(5)
O(1)-Zn(1)-N(3)		129.27(5)	O(4)-Zn(1)-N(4)	97.94(5)
O(1)-Zn(1)-N(4)		105.23(5)	N(3)-Zn(1)-N(4)	99.65(6)
intermolecular interactions (Å)				
D-H···A	D-H (Å)	H···A (Å)	D···A (Å)	>D-H···A (°)
N(1)-H(1)···O(6)	0.88	2.02	2.829(2)	153
N(2)-H(2)···O(3)	0.88	1.99	2.837(2)	160
C(4)-H(4A)···O(6)	0.98	2.49	3.319(2)	142
C(15)-H(15A)···O(3)	0.98	2.33	3.156(2)	142
C(28)-H(28)···O(1W)	0.95	2.59	3.464(3)	154
O(2W)-H(2WO)···O(2)	0.84	1.92	2.727(3)	160
C(2W)-H(2WC)···O(5)	0.98	2.53	3.360(5)	142
C(10)-H(10)···O(3W)	0.95	2.79	3.578(7)	141
C(27)-H(27)···O(2)	0.95	2.45	3.238(3)	140
C(4)-H(4B)···O(4)	0.98	2.55	3.409(2)	147

**Table 3:** Selected Bond Lengths (Å), Bond Angles (°), and Intermolecular Interactions (Å) for **2**^a^

bond lengths (Å)				
Zn(1)-O(1)		1.965(2)	Zn(1)-N(2)	2.022(2)
Bond angles (°)				
O(1)-Zn(1)-O(1)#1		100.30(13)	O(1)-Zn(1)-N(2)#1	104.45(10)
O(1)-Zn(1)-N(2)		114.90(10)	N(2)-Zn(1)-N(2)#1	116.89(14)
intermolecular interactions (Å)				
D-H···A	D-H (Å)	H···A (Å)	D···A (Å)	>D-H···A (°)
N(1)-H(1)···O(2)	0.88	2.04	2.813(3)	146
C(15)-H(15)···O(1)	0.95	2.33	3.167(4)	147
O(1W)-H(1WO)···O(3)	0.84	2.00	2.762(4)	151
C(13)-H(13)···O(1W)	0.95	2.42	3.150(5)	134

a #1: *x* + 1, *y*, −*z* + 3/2.

**Figure 2:**
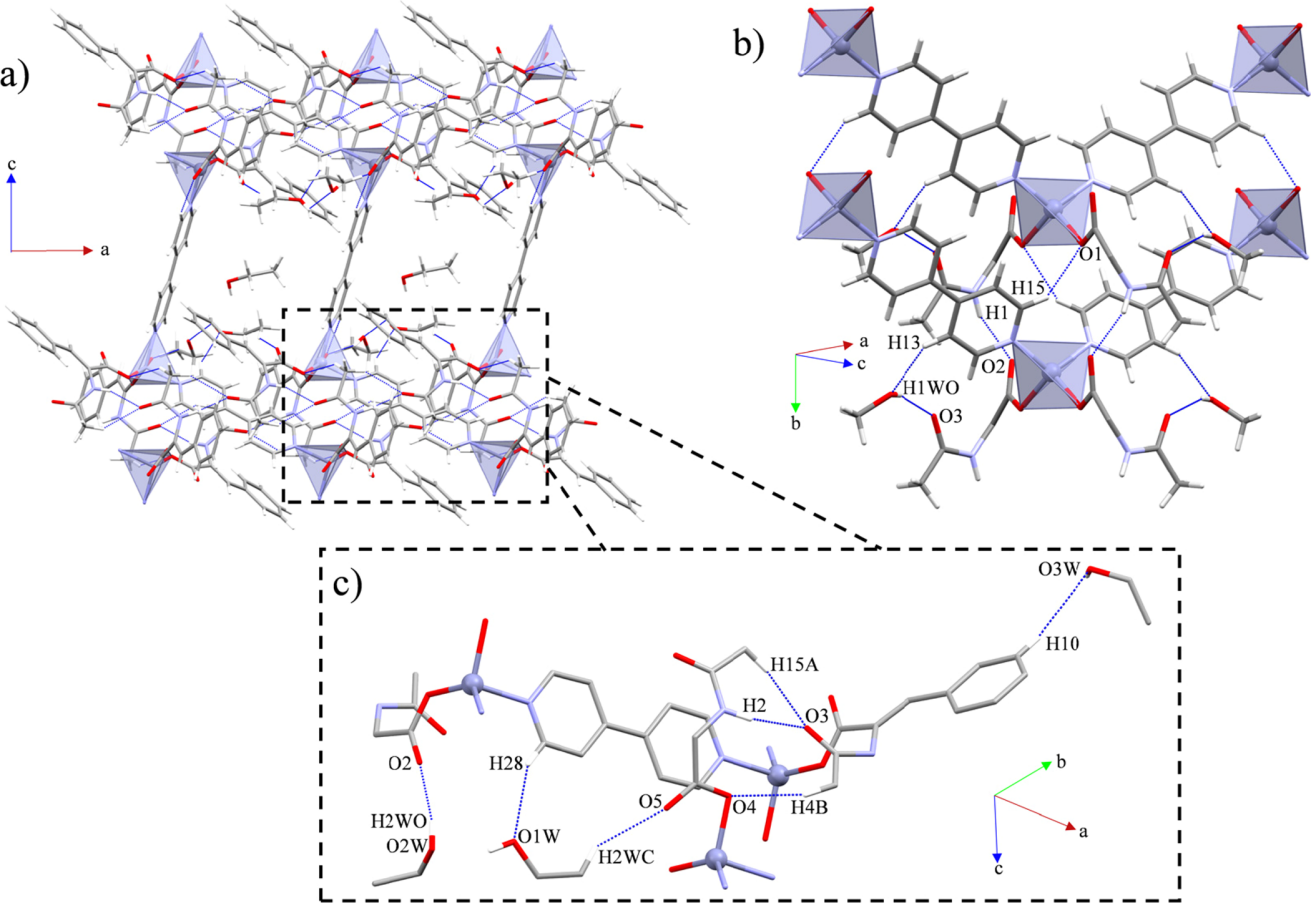
General views of the intermolecular interactions expanding the structure of (a) **1C** through the (101) plane and (b) **2** along the (011) plane. (c) In detail view of the intermolecular interactions of **1C**.

The intermolecular interactions of **1C** and **2** are based on the N–H···O_C=O_ (**1C**) and N–H···O_COO_ (**2**) synthons, both supported by C–H···O interactions involving the methyl groups from ACA and the coordinated carboxylate oxygen atoms and carbonyl oxygen atoms from ACA (**1C**), or the *o*-H atom from 4,4′-bipy and the coordinated oxygen atoms from the carboxylate of ACA (**2**). These interactions expand the structures along the [100] (**1C**) and [010] (**2**) directions, which in combination with the corresponding 4,4′-bipy expansion form 2D supramolecular layers along the (101) (**1C**) and (011) (**2**) planes (Tables 2 and 3; Figure 2a,b). Additionally, one pair of EtOH molecules in **1C** is joined together to the polymeric array through O–H···O interactions with the oxygen atoms from the uncoordinated carboxylate groups, while the other three EtOH molecules present weak C–H···O interactions involving the oxygen atoms from carboxylate ACA groups, a *m*-H from ACA, and a *o*-H from a 4,4′-bipy (Figure 2c). By the same token, the MeOH molecules of **2** interact with the polymeric chains through a double C–H···O interaction involving a *m*-H from 4,4′-bipy and a H-bond between the MeOH and the oxygen atom from the carbonyl moieties of ACA. (Figure 2b).

### Structural Description of {[Zn_2_(μ-ACA)_2_(ACA)_2_(4,4′-bipy)]·2H_2_O}_*n*_ (**3**)

**Figure 3:**
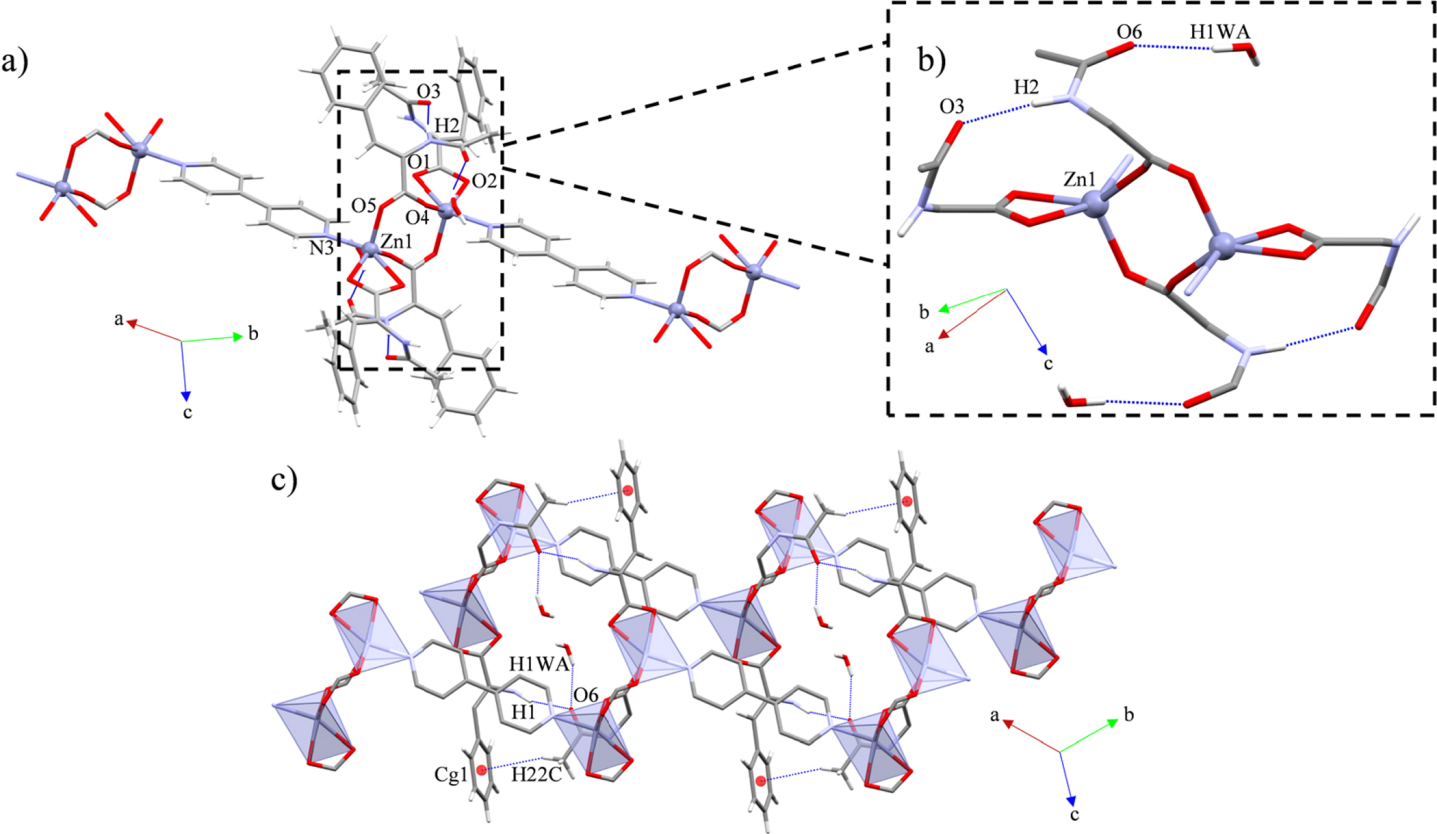
(a) Molecular structure of compound **3**. (b) Intra- and intermolecular interactions involving the amide moieties of ACA. (c) Supramolecular expansion of **3** along the (110) plane.

Compound **3** belongs to the triclinic P1̅ space group. It consists of a *zig-zag* CP expanded along the [11̅0] direction. The CP is formed by dimeric SBUs with a [ZnO_4_N] *core* composed by one bridging (μ_2_-η^1^:η^1^) and one chelate (μ_1_-η^2^) ACA, as well as one 4,4′-bipy ligands, all of them displaying a distorted square pyramidal geometry (S = 2.525) (Figure 3a; SI: Table S1). Furthermore, its τ_5_ value of 0.30 also agrees with a square pyramidal geometry presenting an important distortion.^66^ Additionally, the supramolecular array of **3** generates voids with an accessible volume of 3.10 Å^3^ (0.2% of the unit cell volume),^62^ where two water molecules are located. The bond lengths and bond angles oscillate between 1.9739(11)-2.0750 (13) Å and 61.32(5)-153.20 (5)° (Table 4), presenting similar values than other Zn(II) CPs containing dimeric SBUs formed by carboxylate ligands and 4,4′-bipyridines as spacers.^67−69^

**Table 4:** Selected Bond Lengths (Å), Bond Angles (°), and Intra- and Intermolecular Interactions (Å) for **3**^a^

bond lengths (Å)				
Zn(1)-O(1)		2.2373 (11)	Zn(1)-O(5)#1	1.9755(11)
Zn(1)-O(2)		2.0642(11)	Zn(1)-N(3)	2.0750(13)
Zn(1)-O(4)		1.9739(11)		
bond angles (°)				
O(1)-Zn(1)-O(2)		61.32(5)	O(2)-Zn(1)-O(5)#1	113.45(5)
O(1)-Zn(1)-O(4)		95.34(4)	O(2)-Zn(1)-N(3)	97.29(5)
O(1)-Zn(1)-O(5)#1		108.08(5)	O(4)-Zn(1)-O(5)#1	110.13(4)
O(1)-Zn(1)-N(3)		153.20(5)	O(4)-Zn(1)-N(3)	89.28(5)
O(2)-Zn(1)-O(4)		135.08(5)	O(5)#1-Zn(1)-N(3)	94.91(5)
intramolecular interactions (Å)				
D-H···A	D-H (Å)	H···A (Å)	D···A (Å)	>D-H···A (°)
N(2)-H(2)···O(3)	0.88	1.95	2.8079(16)	166
intermolecular interactions (Å)				
D-H···A	D-H (Å)	H···A (Å)	D···A (Å)	>D-H···A (°)
N(1)-H(1)···O(6)	0.88	2.04	2.8901(17)	161
O(1W)-H(1WA)···O(6)	0.83(3)	2.12(3)	2.900(3)	158(4)
C(22)-H(22C)···Cg(1)	0.98	2.93	3.7856(17)	146

a #1: −*x* + 1, −*y* + 1, −*z* + 1. Cg(1) = C(4) C(5) C(6) C(7) C(8) C(9).

The intramolecular interactions of **3** are based on one N–H···O_C=O_ synthon between contiguous ACA units, which in combination with a O_w_–H_w_···O_C=O_ interaction involving the water molecules support the dimeric array (Figure 3b). Otherwise, their intermolecular interactions consist of weak C–H···π associations involving the methyl groups of ACA and contiguous aromatic rings from nearby ACA ligands, supporting the supramolecular expansion. All this set of interactions expand the structure of **3** along the *a* axis, which in combination with the molecular propagation of the CP forms 2D layers along the (110) plane (Figure 3c).

### Structural Description of {[Zn_3_(μ-ACA)_6_(4,4′-bipy)]·8CHCl_3_}_*n*_ (**4C**)

**Figure 4:**
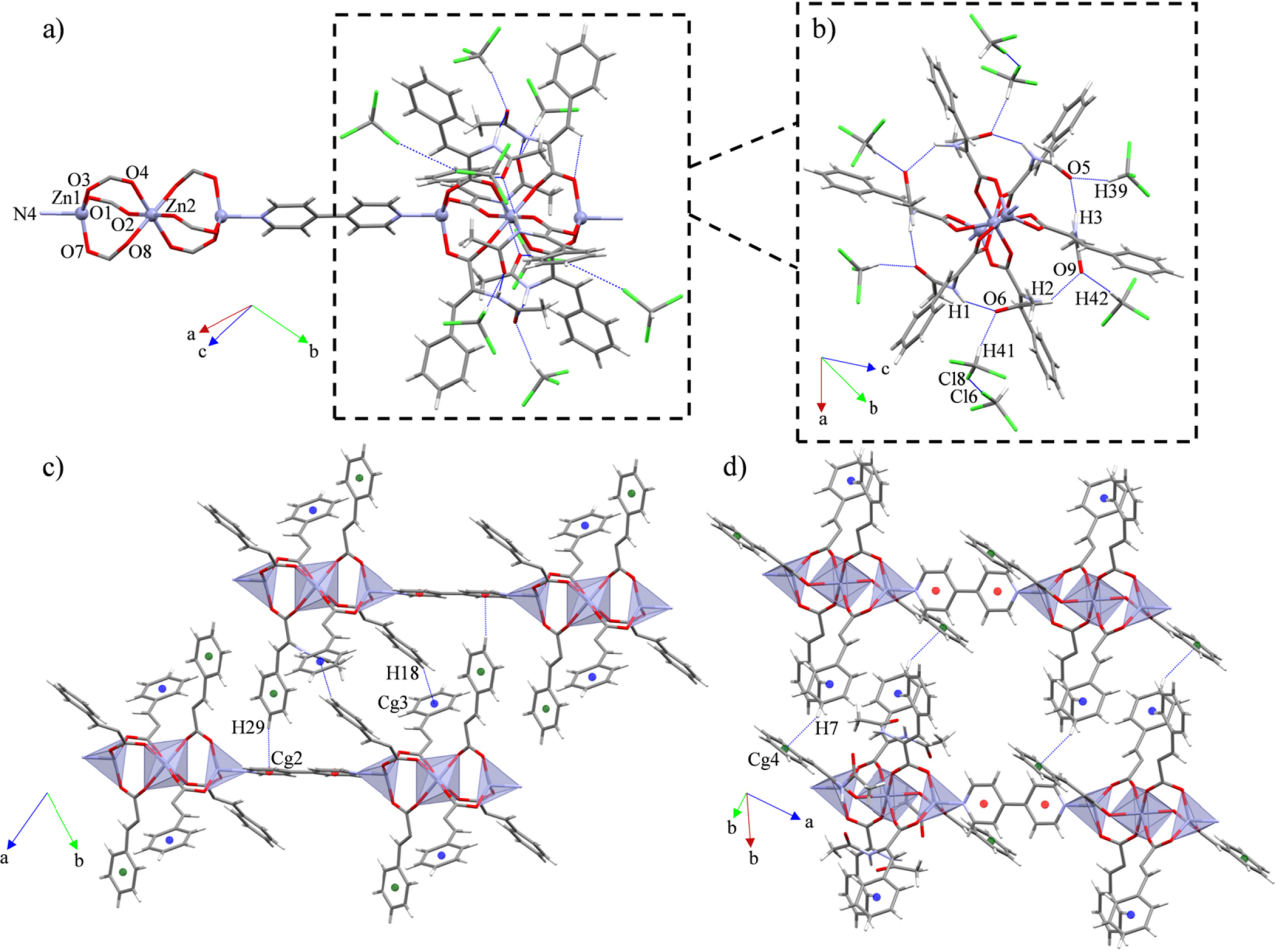
(a) Molecular structure of compound **4C**. (b) Intra- and intermolecular interactions involving the amide moieties of ACA. Supramolecular expansions along the (c) (002) and (d) (110) planes.

Compound **4C** belongs to the triclinic P1̅ space group. It consists of a linear CP formed by pinwheel arrays connected through 4,4′-bipy ligands along the [011̅] direction. The ACA ligands display six μ_2_-η^1^:η^1^ coordination modes, which form the pinwheel SBU, accommodating tetrahedral geometries in the lateral Zn(II) *cores* (S = 0.843, τ_4_ = 0.93),^61^ and an octahedral geometry for the central Zn(II) atom (S = 0.245, *ata* = 60°)^70,71^ (Figure 4a; SI: Table S1). In addition, the supramolecular scaffold of **4C** generates voids with an accessible volume of 406.46 Å^3^ (15.0% of the unit cell volume),^62^ containing eight CHCl_3_ molecules. The bond lengths and bond angles oscillate between 1.926(4)-2.096 (4) Å and 85.08(14)-180° (Table 5), presenting similar values than other Zn(II) CPs bearing pinwheel arrays and 4,4′-bipyridine ligands as spacers.^52,72,73^

**Table 5:** Selected Bond Lengths (Å), Bond and Twist Angles (°), and Intra- and Intermolecular Interactions (Å) for **4C**

bond lengths (Å)				
Zn(1)-O(1)		1.941(4)	Zn(2)-O(2)	2.065(4)
Zn(1)-O(2)		2.065(4)	Zn(2)-O(4)	2.089(3)
Zn(1)-O(3)		1.926(4)	Zn(2)-O(8)	2.096(4)
Zn(1)-N(4)		2.046(4)		
bond angles (°)				
O(1)-Zn(1)-O(3)		114.35(17)	O(2)-Zn(2)-O(4)#1	85.90(15)
O(1)-Zn(1)-O(7)		114.80(17)	O(2)-Zn(2)-O(8)	92.32(15)
O(1)-Zn(1)-N(4)		102.29(17)	O(2)-Zn(2)-O(8)#1	87.68(15)
O(3)-Zn(1)-O(7)		122.55(17)	O(4)-Zn(2)-O(4)#1	180.0
O(3)-Zn(1)-N(4)		97.29(17)	O(4)-Zn(2)-O(8)	94.92(14)
O(7)-Zn(1)-N(4)		99.58(17)	O(4)-Zn(2)-O(8)#1	85.08(14)
O(2)-Zn(2)-O(2) #1		180.0	O(8)#1-Zn(2)-O(8)	180.0
O(2)-Zn(2)-O(4)		94.11(15)		
twist angles (°)				
O(2)-Cg(1)-Cg(1)-O(8)		61.31	O(8)-Cg(1)-Cg(1)-O(4)	58.66
O(4)-Cg(1)-Cg(1)-O(2)		60.03		
intramolecular interactions (Å)				
D-H···A	D-H (Å)	H···A (Å)	D···A (Å)	>D-H···A (°)
N(1)-H(1)···O(6)	0.88	2.04	2.811(6)	146
N(2)-H(2)···O(9)	0.88	2.22	2.908(6)	135
N(3)-H(3)···O(5)	0.88	1.99	2.850(7)	166
intermolecular interactions (Å)				
D-H···A	D-H (Å)	H···A (Å)	D···A (Å)	>D-H···A (°)
C(39)-H(39)···O(5)	1.00	2.06	3.01(1)	157
C(41)-H(41)···O(6)	1.00	2.06	3.023(7)	161
C(38)-H(38)···Cl(7)	0.95	2.89	3.812(5)	165
C(42)-H(42)···O(9)	1.00	2.16	3.142(9)	167
C(29)-H(29)···Cg(2)	0.95	2.75	3.628(8)	154
C(18)-H(18)···Cg(3)	0.95	2.59	3.431(8)	148
C(7)-H(7)···Cg(4)	0.95	2.83	3.636(8)	143
halogen bonds				
C-Cl···Cl	C-Cl (Å)	Cl···Cl (Å)	θ^a^ (°)	
C(41)-Cl(8)···Cl(6)	1.722(7)	3.391(3)	152.1(3)	
C(40)-Cl(6)···Cl(8)	1.732(7)		167.6(3)	

a θ = C-Cl···Cl angle. Cg(1) = O(2) O(4) O(8); Cg(2) = N(4) C(34) C(35) C(36) C(37) C(38); Cg(3) = C(4) C(5) C(6) C(7) C(8) C(9); Cg(4) = C(26) C(27) C(28) C(29) C(30) C(31).

The intramolecular interactions of **4C** consist of a hexagonal pattern of contiguous N–H···O_C=O_ synthons that stabilize the pinwheel array (Figure 4b). By the other side, its intermolecular interactions show that six CHCl_3_ molecules are placed around the polymeric chains occupying all the carbonyl oxygen atoms from the ACA ligands forming C–H···O interactions. In addition, two CHCl_3_ molecules are joined together to nearby CHCl_3_, displaying quasi-type I halogen bond interactions (|θ_1_ – θ_2_| = 15.53^°^) (Figure 4b).^74^ Because the strong H-bonds partake in the intramolecular stabilization of the SBU and the interactions with the solvent molecules, the supramolecular expansion of the chain is performed by three weak C–H···π interactions between ACA and 4,4′-bipy ligands and ACA ligands by itself expanding the structure along the (002) and (110) planes (Figure 4c,d). All this set of supramolecular interactions combined with the propagation of the CP form a 3D net.

### Solvent Controlled SBU Formation

The intricated energetic landscape of CPs allows the obtention of different molecular arrays with subtle energetic differences that can be overcome by supramolecular interactions (e.g., H-bonds or planar interactions), guiding toward specific assemblies.^75^ For accomplishing that, hydrogen bonding has been used as an important structure-directing agent for the obtention of CPs. In this context, the role of amide-based linkers driving the formation of different CPs has been widely reported, yielding structural transformations based on the reorganization of the amide groups leading to different linker conformations.^76−79^ However, examples of structural transformations producing changes on the SBU caused by amide groups have been scarce.^80^ These functional groups tend to form amide-amide self-complementary interactions, unless they present geometrical restrictions or competing donor/acceptor groups such as solvent molecules, which can disrupt the amide-amide homosynthons.^81^ Regarding ACA-based complexes, the strong influence of solvent molecules on the behavior of their amide groups has been previously observed by our group, showing that this effect can lead to complexes with different SBUs.^51−54^ Herein, the formation of CPs with controlled SBUs depending on the utilized solvents has been discussed.

**Figure 5:**
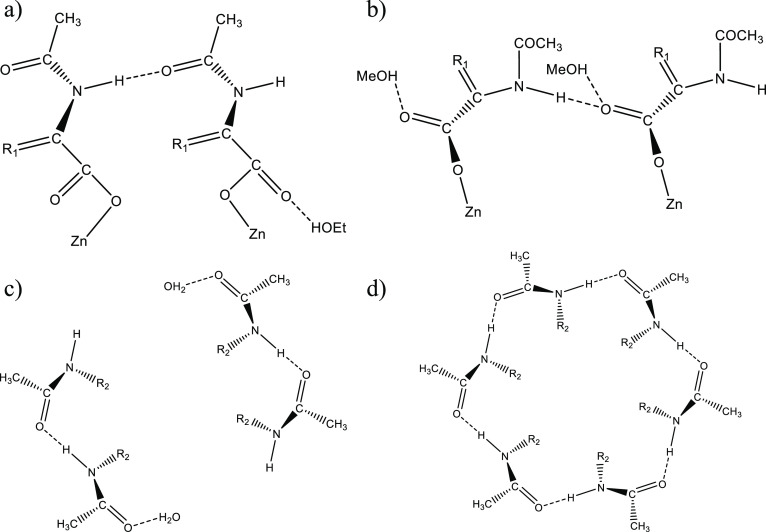
Amide behavior in the crystal packing of (a) **1C**, (b) **2**, (c) **3**, and (d) **4C**. R_1_ stands for C_7_H_6_ while R_2_ for C_9_H_6_O_2_.

Compounds **1C** and **2** present two uncoordinated carboxylate oxygen atoms able to competitively disrupt the amide···amide homosynthon. In **1C**, the amide moieties display self-complementary intermolecular interactions and the EtOH molecules form additional H-bonds with uncoordinated carboxylate oxygen atoms from ACA (Figure 5a). Otherwise, in compound **2**, their two uncoordinated carboxylate groups interact with the NH of the amide moieties, while the MeOH molecules are joint to the C=O groups (Figure 5b). This behavior could be ascribed to the slightly better H-donor propensity of MeOH (Σα = 0.43) compared with EtOH (Σα = 0.37),^55^ conjointly with the possibility of the combination of the N–H···O_COO_ and the MeOH···O_COO_ interactions to compete with the amide-amide homosynthons,^82,83^ promoting that the amide moieties fold outward from ACA to interact with the MeOH molecules in **2**.

For compound **3**, the amide moieties display self-complementary interactions as in **1C**, but in this case, the ACA ligands slightly fold inward in pairs toward the formation of intramolecular amide-amide homosynthons. The increase of temperature utilized for reaching **3** from **1** could be an important factor that provides enough energy to fold the SBU, forming an intramolecular pattern that is disrupted by the incorporation of water molecules that replace the position of two amide groups. These water molecules can form H-bonds with interaction energies lying in the same range than the amide-amide interactions (|*E*_int_| = 35–40 KJ/mol),^84^ being able to disrupt the pattern of amide-amide homosynthons, which does not allow additional ACA ligands to partake in them, and thus, the trimeric pinwheel array cannot be formed, yielding dimeric SBUs (Figure 5c).

**Figure 6:**
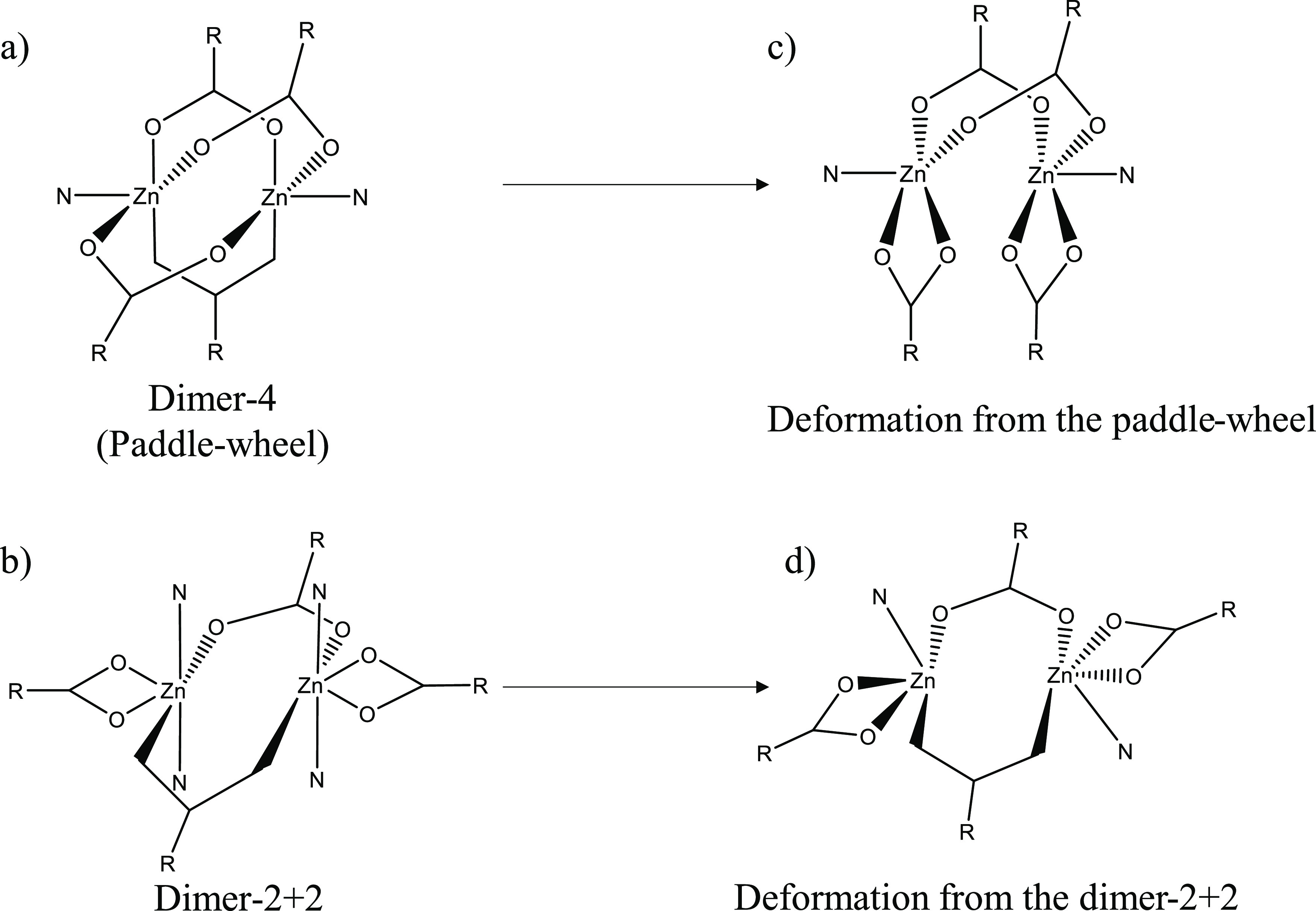
Structure of (a) dimer-4 (paddle-wheel), (b) dimer-2 + 2, (c) deformation from the paddle wheel, and (d) deformation from the dimer-2 + 2.

A search in the Cambridge Structural Database (CSD)^85^ of CPs containing Zn(II) dimeric SBUs with two bridging and two chelate coordination modes of the carboxylate groups as well as pyridine derivatives resulted in 18 hits. This criterion was applied in order to exclude the most common paddle-wheel (dimer-4) arrays from the search, which present four bridging coordination modes (Figure 6a).^86^ From this search, four different *cores* have been found, displaying five hits with [ZnO_4_N_2_], four hits with [ZnO_5_N], eight hits with [ZnO_4_N], and one hit with [ZnO_3_N] *cores*. All of the structures presenting [ZnO_4_N_2_] *cores* display the dimeric-2 + 2 array (Figure 6b), while the other three types of arrangements are divided into the paddle-wheel and the dimer-2 + 2 deformations (Figure 6c,d). J.J. Vittal group reported a rare deformation of the paddle-wheel SBU,^87^ which has been found in eight structures (four with [ZnO_4_N], three with [ZnO_5_N], and one with [ZnO_3_N] *cores*). All of them have been found to be stabilized by planar intramolecular interactions unless two examples where a double intermolecular C–H···O interaction stabilizes the distortion (Figure 6c). By the other side, the distortion from the dimer-2 + 2 has been less reported with only three examples found in the literature (one with [ZnO_5_N]^88^ and two with [ZnO_4_N]^89,90^*cores*) (Figure 6d). From them, it has been noticed that both examples showing [ZnO_4_N] *cores* are stabilized by N–H···O intramolecular interactions such in **3**, which promotes a nearing in pairs of the carboxylate ligands, leading to the cleavage of a pair of pyridine ligands (one per metal atom) owing to the lack of available space in the coordination sphere. For compound **3**, the dimeric SBU disposition leads to the uncommon distortion from the dimer-2 + 2 supported by amide-amide intramolecular homosynthons, being in line with the two previous examples containing [ZnO_4_N] *cores.*(89,90) Thus, the combination of the amide-amide homosynthon that supports the dimeric SBU formation, combined with the presence of water molecules that disrupt the closing of the round hexagonal pattern of amide-amide intramolecular homosynthons, outstand as two important factors for the obtention of this uncommon distortion of the dimer-2 + 2 SBU in **3** (Figure 6d).

Conversely, the recrystallization of **1** in CHCl_3_ results in the folding inward of the amide moieties forming a pinwheel array caused by a round hexagonal pattern of amide-amide intramolecular homosynthons (**4C**). Indeed, the presence of CHCl_3_ as the solvent, which does not compete with the homosynthon formation owing to their poorer H-donor propensity (Σα = 0.15),^55^ allows to bring together three Zn units completing the round hexagonal pattern of amide-amide homosynthons (Figure 5d).

A CSD search of CPs with the pinwheel array containing 4,4′-bipyridine ligands reveal a total of 22 hits.^85^ From them, five structures are not directed by supramolecular interactions that favor the pinwheel SBU, while 17 structures are stabilized by round patterns formed by strong H-bonds (seven structures) or planar interactions (seven structures), and three structures stabilize the array through weak C–H···O associations. Hence, patterns such as the round hexagonal pattern of amide-amide homosynthons arise as an important stabilizing agent for the obtention of pinwheel SBUs.

### Interconversions between **1**–**4**

**Scheme 2:**
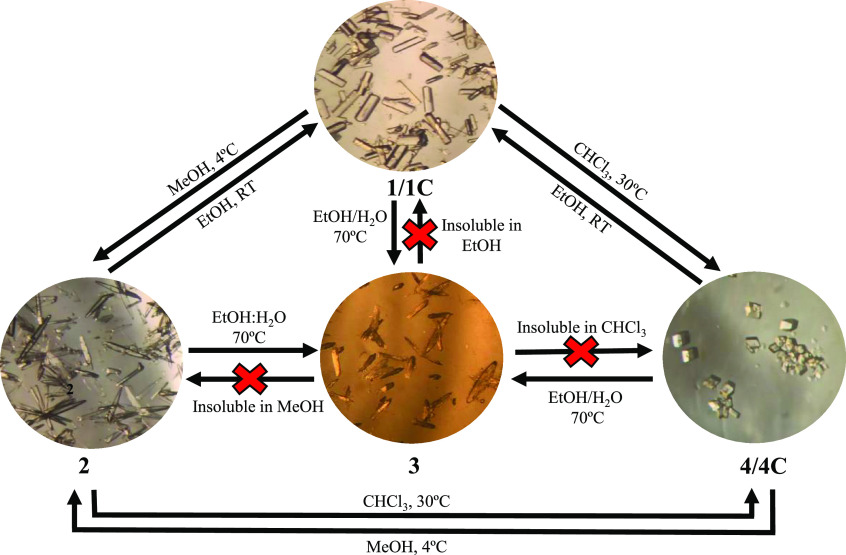
Single Crystals of **1/1C**-**4**/**4C** with an Overview of the Proper Conditions To Achieve the Corresponding Structural Transformations

The reversibility of the structural transformations to compound **1**, as well as the interconversion between **2**–**4**, has been assayed and followed by PXRD and FTIR-ATR (SI: Figures S2–S5 and S9–S12). Compounds **2** and **4** can be successfully converted to **1** after dissolution in EtOH at RT during 12 h. Furthermore, they can be transformed one into another reversibly (**2**↔**4**) using the same synthetic conditions as for the obtention of **2** and **4** from **1** (see the Experimental Section), and both can be interconverted to **3** (**2** → **3** and **4** → **3**) when the same synthetic conditions as for the transformation of **1** → **3** were performed (see the Experimental Section). Conversely, compound **3** cannot be transformed to neither **1**, **2**, and **4** because it does not present enough solubility in MeOH, EtOH, and CHCl_3_ to perform the DRSTs (Scheme 2). Therefore, these results show that when dissolution is possible, the amide moieties of ACA orientate inward or outward, driving the formation CPs with specific arrays that are highly dependent on the solvent (**1**, **2**, and **4**) or both the solvent and temperature (**3**).

### Photoluminescence

**Figure 7:**
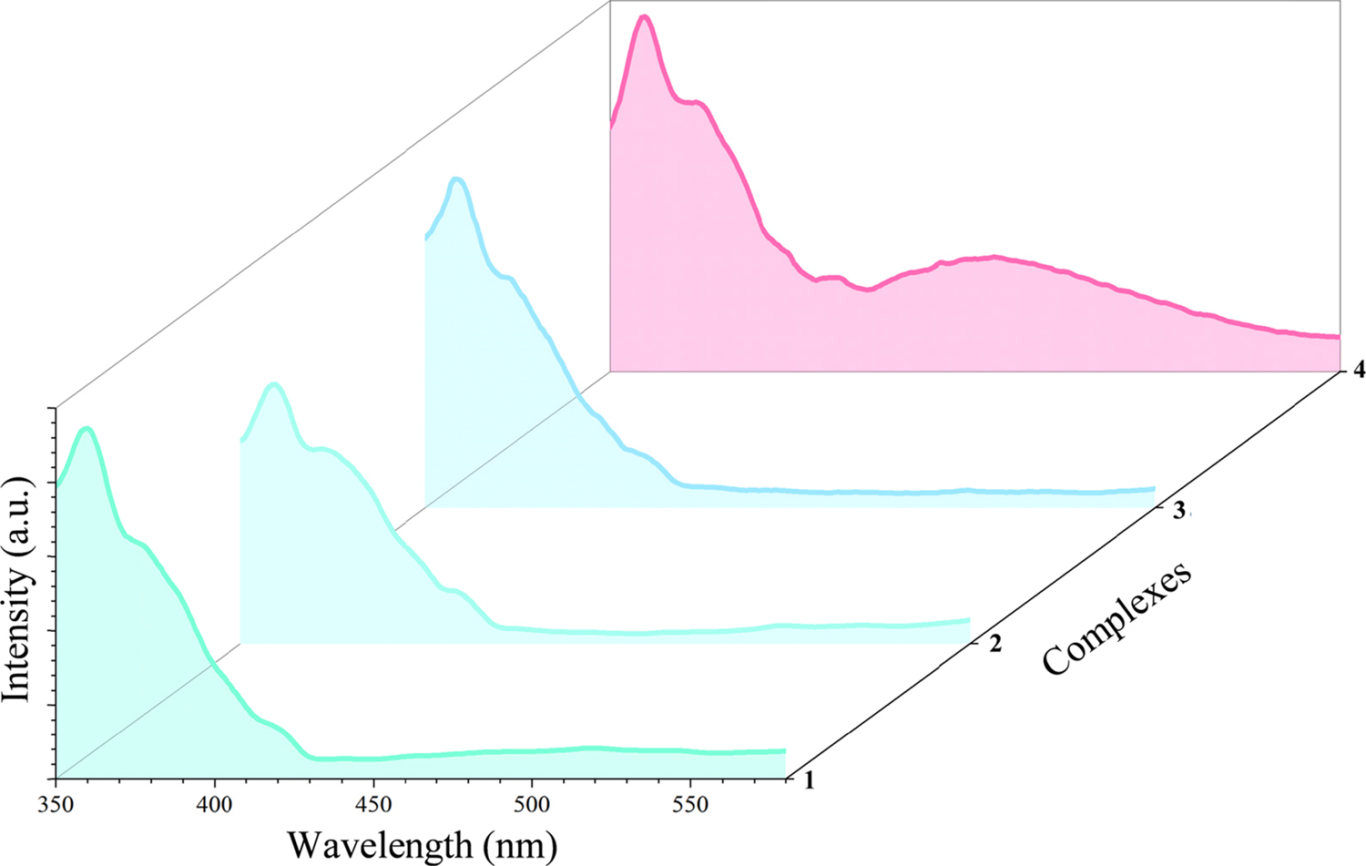
Solid-state emission spectra of compounds **1**–**4** excited at λ_exc_ = 320 nm. The selected colors under the curves of each compound correspond to the observed colors when irradiated at 320 nm according to the CIE 1931 chromaticity diagram.

Photoluminescence properties of **1**–**4** were recorded using UV excitation of a pulse laser beam at λ_exc_ = 320 nm. Under irradiation, the CPs show unstructured emission signals suggesting charge transfer transitions (Figure 7).^91^ All the emission spectra present one common emission maxima (λ_max-em_) centered at 361 nm with a Stokes shift of 3549 cm^–1^. This band could be assigned to the 4,4′-bipy ligand because it emits in this region when excited at 323 nm.^92^ Interestingly, compound **4** presents an additional band at 469 nm with a Stokes shift of 9928 cm^–1^. It has been noticed that the presence of Stokes shifts larger than 8000 cm^–1^ is associated with excited-state proton transfers (ESPT).^93^ These processes typically take place between atoms involved in strong H-bonds usually bearing -OH functionalities or less commonly -NHR groups as H-donors, which allow their rational functionalization with electron-donor/-withdrawing groups, resulting in tunable emission wavelengths.^94−96^ In **4**, the interaction of the amide moieties is reinforced by the incorporation of the electron-withdrawing acetyl moieties, which increased the acidity of the N–H groups and thus drove the formation of the amide-amide round hexagonal pattern of amide-amide homosynthons. Bearing these factors in mind, compound **4** presents the proper structural features, and thus, the additional band on their emission spectrum has been ascribed to an ESPT between amides moieties themselves. Otherwise, the arrangement of the amide moieties in **1**–**3** seems to not favor the proton transfer between these amide groups, which may explain that the band of 469 nm is not present in these CPs. The resultant emission colors for **1**–**4** under irradiation at 320 nm are aquamarine (**1**), frosted mind (**2**), anakiwa (**3**), and hot pink (**4**), in accordance with the CIE 1931 chromaticity diagrams (Figure 7; SI: Figure S21),^97^ which remarks the difference in color between **1**–**3** and **4**. Therefore, the photoluminescence measurements of this series of CPs, all of them bearing the same former ligands but with different molecular arrays, show how differences on the molecular structure have their effect on the photoluminescence properties, these two factors being directly correlated.

## Conclusions

We have synthesized one CP (**1**) used as a starting compound for the obtention of three CPs with variable SBUs (**2**–**4**) through DRST processes. It has been shown that the amide orientation in the presence of different solvents plays a key role on the stabilization of specific SBUs, allowing the obtention of monomeric (**1**, **2**), dimeric (**3**), or trimeric (**4**) SBUs depending on the utilized solvent and/or the temperature. Interestingly, the presence of water in the DRST of **3** has disrupted the round hexagonal pattern of amide–amide homosynthons driving the formation of an uncommon dimeric array coming from the deformation of the dimeric-2 + 2 SBU. Furthermore, the round hexagonal pattern of amide–amide homosynthons of **4C** has also been shown as a good stabilizing agent for the obtention of pinwheel arrays. Interconversion assays demonstrate that these transformations are reversible in most of the cases, unless for compounds **3**, which could be attributed to its low solubility in MeOH, EtOH, and CHCl_3_ that do not allow the reorganization of the polymeric array through the corresponding DRST. Finally, solid-state photoluminescence has been measured observing a different behavior of **4** compared with **1**–**3**, which may be attributed to an ESPT process involving the round hexagonal pattern of amide–amide homosynthons that form its pinwheel array. Therefore, this work contributes to the understanding of structure–property correlations in CPs and provide an example of how CPs consisting of the same former ligands with different molecular arrays present differences in their photoluminescent properties.

## Supporting Information Available

Further details of the cooling ramp utilized for the obtention of single crystals of **1C**, PXRD diffractograms, TG-DTA of **1** and **4**, HR-ESI-MS, FTIR-ATR, ^1^H NMR, ^13^C{^1^H} NMR, DEPT-135 NMR spectra of **1**–**4**, as well as relevant data for the geometric evaluations of **1C**, **2**, **3**, and **4C***cores* and photoluminescence properties of **1**–**4** (PDF)
