# Supplementary material for: Amide-Driven Secondary Building Unit Structural Transformations between Zn(II) Coordination Polymers
Source: Cryst Growth Des. 2022 Jul 13;22(8):5012–26. doi: 10.1021/acs.cgd.2c00520 (PMC9374304; doi:10.1021/acs.cgd.2c00520)
Supplement: Supplementary file 1 — cg2c00520_si_001.pdf [file cg2c00520_si_001.pdf]

# Supporting Information

# Amide-driven secondary building unit structural transformations between Zn(II) coordination polymers

*Daniel Ejarque [a], Teresa Calvet [b], Mercè Font-Bardia [c], and Josefina Pons [a,*]*

[a]Departament de Química, Universitat Autònoma de Barcelona, 08193-Bellaterra, Barcelona, Spain

[b]Departament de Mineralogia, Petrologia i Geologia Aplicada, Universitat de Barcelona, Martí i Franquès s/n, 08028 Barcelona, Spain

[c]Unitat de Difracció de Raig-X, Centres Científics i Tecnològics de la Universitat de Barcelona (CCiTUB), Universitat de Barcelona, Solé i Sabarís, 1-3, 08028 Barcelona, Spain

*Corresponding author E-mail: josefina.pons@uab.es

# EXPERIMENTAL SECTION

## Materials and general details

Zinc(II) acetate dihydrate ($Zn(OAc)_2·2H_2O$), α-acetamidocinnamic acid (HACA), 4,4'-bipyridine (4.4'-bipy) ligands, and anhydrous ethanol (EtOH), chloroform ($CHCl_3$), diethyl ether ($Et_2O$) and methanol (MeOH) as solvents were purchased from Sigma-Aldrich. The water used for the obtention of **3** was MilliQ. Deuterated dimethylsulfoxide (DMSO-$d_6$) was used for the NMR experiments and was purchased from Eurisotop. All of them were used without further purification. The reactions and manipulations for the obtention of **1** and **2** were carried out at room temperature (RT), while those for the obtention of **1C**, **3** and **4C** were done in a Digiheat-TFT furnace (JP Selecta) using sealed vials under autogenous pressure using a cooling ramp from 50ºC to 25ºC (**1C**), or a constant temperature of 70 ºC (**3**), or 30 ºC (**4C**). Compound **4** was obtained after air exposure of **4C**. Powder X-ray diffraction (PXRD) patterns were measured with a Siemens D5000 apparatus with 40 kW and 45 mA using CuKα radiation with λ = 1.5406 Å. All of them were recorded from 2Θ = 5º to 30º with a step scan of 0.02º counting one second at each step. Elemental analyses (C, H, N) were carried on a Thermo Scientific Flash 2000 CHNS Analyzer. Simultaneous thermogravimetric (TG)/differential thermal analysis (DTA) determinations for **1** and **4** were performed using 57.6 mg (**1**) and 53.5 mg (**4**) in a Netzsch STA 409 instrument, with an aluminum oxide powder crucible and an oxide powder as a standard ($Al_2O_3$, PerkinElmer 0419-0197), and heating at 5 $°C·min^{-1}$ from 25 to 350 °C, under nitrogen atmosphere with a flow rate of 80 $mL·min^{-1}$. HR-ESI-MS measurements were recorded after dissolving the corresponding complexes in MeOH in a MicroTOF-Q instrument equipped with an electrospray ionization source (ESI) in positive mode. $Na^+$ ions come from the MeOH solvent which contains <50 ppb. Conditions were those used in routine experiments. The nebulizer pressure was 1.5 bar, the desolvation temperature was 180 ºC, dry gas at 6 $L·min^{-1}$, the capillary counter-electrode voltage was 5 kV, and the quadrupole ion energy, 5.0 eV. FTIR-ATR spectra were recorded on a Perkin Elmer spectrometer, equipped with an attenuated total reflectance (ATR) accessory model MKII Golden Gate with diamond window in the range 4000–500 $cm^{-1}$. $^1H$, $^{13}C\{^1H\}$ and DEPT-135 NMR spectra were recorded on a Bruker Ascend 300 MHz spectrometer in DMSO-$d_6$ solutions at RT. All the chemical shifts (δ) are given in ppm relative to TMS as internal standard. Solid-state photoluminescence measurements were recorded using a Varian Cary Eclipse

Fluorescence spectrophotometer between 350 and 600 nm. CIE 1931 chromaticity diagram was generated using Origin Pro 2019b software.

**Characterization of compounds 1-4**

**{[Zn(ACA)$_2$(4,4'-bipy)]·EtOH}$_n$ (1).** Yield: 262 mg (85.1%) (based on Zn). Elemental analysis calc(%). for $C_{34}H_{34}ZnN_4O_7$ (676.05): C 60.40; H 5.07; N 8.29; found: C 60.28; H 5.15; N 8.17. HR-MS (ESI$^+$, MeOH): m/z (%) = 157.0768 (100%) (calc. for [4,4'-bipy + H$^+$] = 157.0760); 228.0630 (100%) (calc. for [HACA + Na]$^+$ = 228.0631); 473.0697 (100%) (calc. for [Zn(ACA)$_2$ + H]$^+$ = 473.0686); 495.0492 (100%) (calc. for [Zn(ACA)$_2$ + Na]$^+$ = 495.0505); 740.0591 (79%) (calc. for [Zn$_2$(ACA)$_3$]$^+$ = 740.0559); 967.1126 (74%) (calc. for [Zn$_2$(ACA)$_4$ + Na]$^+$ = 967.1118); 504.0268 (40%) (calc. for [Zn$_3$(ACA)$_4$]$^{2+}$ = 504.0253). FTIR-ATR (wavenumber, cm$^{-1}$): 3455-3378(br) [ν(O-H)], 3237(w) [ν(N-H)], 3163-3023(br) [ν(C-H)$_{ar}$] + [ν(C-H)$_{alk}$], 2980-2946(br) [ν(C-H)$_{al}$], 1661(w) [ν(C=O)], 1642(sh), 1608(s) [$\nu_{as}$(COO)], 1519(m) [ν(C=C/C=N)], 1491(m) [ν(C=C/C=N)], 1447(w), 1417(m), 1372(s) [$\nu_s$(COO)], 1340(s) [δ(C=C/C=N)], 1283(m), 1222(w), 1216(sh), 1182(w), 1144(w), 1072(w) [$\delta_{ip}$(C-H)], 1045(w), 1032(w), 1017(w) [$\delta_{ip}$(C-H)], 985(w), 927(w), 891(w), 850(w), 813(w), 775(w), 769(w), 744(w), 731(w), 692(s) [$\delta_{oop}$(C-H)], 640(m) [$\delta_{oop}$(C-H)], 601(sh), 592(m), 571(m), 554(w), 525(m). $^1$H NMR (300 MHz; DMSO-$d_6$; Me$_4$Si; 298 K): δ = 9.20 [2H, s, N$H_{ACA}$], 8.73 [4H, dd, $^3$J = 4.6 Hz, $^4$J = 1.6 Hz, *o*-$H_{4,4'\text{-bipy}}$], 7.85 [4H, dd, $^3$J = 4.5 Hz, $^4$J = 1.7 Hz, *m*-$H_{4,4'\text{-bipy}}$], 7.50 [4H, d, $^3$J = 7.2 Hz, *o*-$H_{ACA}$], 7.35 [4H, t, $^3$J = 7.3 Hz, *m*-$H_{ACA}$], 7.29 [2H, d, $^3$J = 7.1 Hz, *p*-$H_{ACA}$], 7.24 [2H, s, NH-C-C$H_{ACA}$], 1.95 [6H, s, CO-C$H_{3,ACA}$]. $^{13}$C{$^1$H} NMR (75 MHz; DMSO-$d_6$; Me$_4$Si; 298 K): δ = 170.6 [NH-$C$O$_{ACA}$], 168.7 [$C$OO$_{ACA}$], 150.7 [*o*-$C_{4,4'\text{-bipy}}$], 144.7 [N-CH-CH-$C_{4,4'\text{-bipy}}$], 135.1 [O$_2$C-$C_{ACA}$], 129.7 [HN-C-CH-$C_{ACA}$], 129.4 [*o*-$C_{ACA}$], 129.3 [*p*-$C_{ACA}$], 128.5 [*m*-$C_{ACA}$], 128.4 [NH-C-$C$H$_{ACA}$], 121.6 [*m*-$C_{4,4'\text{-bipy}}$], 23.1 [CO-$C$H$_{3,ACA}$]. DEPT-135 NMR (75 MHz; DMSO-$d_6$; Me$_4$Si, 298 K): δ = 150.7 [*o*-$C_{4,4'\text{-bipy}}$], 129.4 [*o*-$C_{ACA}$], 129.3 [*p*-$C_{ACA}$], 128.5 [*m*-$C_{ACA}$], 128.4 [NH-C-$C$H$_{ACA}$], 121.6 [*m*-$C_{4,4'\text{-bipy}}$], 23.1 [CO-$C$H$_{3,ACA}$].

**{[Zn(ACA)$_2$(4,4'-bipy)]·2MeOH}$_n$ (2).** Yield: 11.6 mg (56.5%) (based on Zn). Elemental analysis calc(%). for $C_{34}H_{36}ZnN_4O_8$ (694.06): C 58.84; H 5.23; N 8.07; found: C 58.58; H 4.98; N 7.92. HR-MS (ESI$^+$, MeOH): m/z (%) = 157.0768 (100%) (calc. for [4,4'-bipy + H]$^+$ = 157.0760); 228.0630 (100%) (calc. for [HACA + Na]$^+$ = 228.0631); 473.0697 (100%) (calc. for [Zn(ACA)$_2$ + H]$^+$ = 473.0686); 495.0492 (100%) (calc. for [Zn(ACA)$_2$ + Na]$^+$ = 495.0505); 740.0591 (79%) (calc. for [Zn$_2$(ACA)$_3$]$^+$ = 740.0559);

967.1126 (74%) (calc. for $[Zn_2(ACA)_4 + Na]^+$ = 967.1118); 504.0268 (40%) (calc. for $[Zn_3(ACA)_4]^{2+}$ = 504.0253). FTIR-ATR (wavenumber, $cm^{-1}$): 3646(w) [ν(O-H)], 3403(w), 3248(w) [ν(N-H)], 3171-3025(br) [$\nu(C\text{-}H)_{ar}$] + [$\nu(C\text{-}H)_{alk}$], 2999-2812(br) [$\nu(C\text{-}H)_{al}$], 1668(w) [ν(C=O)], 1643(w), 1613(sh), 1593(s) [$\nu_{as}(COO)$], 1515(m) [ν(C=C/C=N)], 1491(m) [ν(C=C/C=N)], 1447(w), 1422(m), 1385(s) [$\nu_s(COO)$], 1370(s), 1355(s) [δ(C=C/C=N)], 1328(br), 1275(m), 1269(w), 1223(w), 1208(w), 1187(w), 1149(w), 1070(w) [$\delta_{ip}(C\text{-}H)$], 1032(w) [$\delta_{ip}(C\text{-}H)$], 1017(w) [$\delta_{ip}(C\text{-}H)$], 1003(w), 987(w) [$\delta_{ip}(C\text{-}H)$], 924(w), 896(w), 851(w), 818(m), 772(m), 750(m), 727(w), 688(s) [$\delta_{oop}(C\text{-}H)$], 641(m) [$\delta_{oop}(C\text{-}H)$], 629(sh), 594(m), 556(m), 523(m). $^1H$ NMR (300 MHz; DMSO-$d_6$; $Me_4Si$; 298 K): δ = 9.17 [2H, s, $NH_{ACA}$], 8.72 [4H, dd, $^3J$ = 4.5 Hz, $^4J$ = 1.7 Hz, $o\text{-}H_{4,4'\text{-bipy}}$], 7.83 [4H, dd, $^3J$ = 4.5 Hz, $^4J$ = 1.7 Hz, $m\text{-}H_{4,4'\text{-bipy}}$], 7.49 [4H, d, $^3J$ = 7.4 Hz, $o\text{-}H_{ACA}$], 7.34 [4H, t, $^3J$ = 7.3 Hz, $p\text{-}H_{ACA}$], 7.28 [2H, d, $^3J$ = 7.2 Hz, $m\text{-}H_{ACA}$], 7.23 [2H, s, NH-C-$CH_{ACA}$], 4.18 [2H, q, $^3J$ = 5.1 Hz, $OH_{MeOH}$], 3.16 [6H, d, $^3J$ = 5.1 Hz, $CH_{3,MeOH}$], 1.95 [6H, s, CO-$CH_{3,ACA}$]. $^{13}C\{^1H\}$ NMR (75 MHz; DMSO-$d_6$; $Me_4Si$; 298 K): δ = 170.3 [NH-$CO_{ACA}$], 168.5 [$COO_{ACA}$], 150.7 [$o\text{-}C_{4,4'\text{-bipy}}$], 144.6 [N-CH-CH-$C_{4,4'\text{-bipy}}$], 135.2 [$O_2C\text{-}C_{ACA}$], 129.8 [HN-C-CH-$C_{ACA}$], 129.4 [$o\text{-}C_{ACA}$], 128.8 [$p\text{-}C_{ACA}$], 128.4 [$m\text{-}C_{ACA}$], 128.2 [NH-C-$CH_{ACA}$], 121.5 [$m\text{-}C_{4,4'\text{-bipy}}$], 48.7 [$CH_3OH$], 23.1 [CO-$CH_{3,ACA}$]. DEPT-135 NMR (75 MHz; DMSO-$d_6$; $Me_4Si$, 298 K): δ = 150.7 [$o\text{-}C_{4,4'\text{-bipy}}$], 129.4 [$o\text{-}C_{ACA}$], 128.9 [$p\text{-}C_{ACA}$], 128.4 [$m\text{-}C_{ACA}$], 128.2 [NH-C-$CH_{ACA}$], 121.5 [$m\text{-}C_{4,4'\text{-bipy}}$], 48.7 [$CH_3OH$], 23.1 [CO-$CH_{3,ACA}$].

**$\{[Zn_2(\mu\text{-}ACA)_2(ACA)_2(4,4'\text{-bipy})]\cdot 2H_2O\}_n$ (3).** Yield: 9.3 mg (55.2%) (based on Zn). Elemental analysis calc(%). for $C_{54}H_{52}Zn_2N_6O_{14}$ (1139.80): C 56.90; H 4.60; N 7.37; found: C 56.74; H 4.48; N 7.20. HR-MS ($ESI^+$, MeOH): m/z (%) = 157.0768 (100%) (calc. for $[4,4'\text{-bipy} + H]^+$ = 157.0760); 228.0630 (100%) (calc. for $[HACA + Na]^+$ = 228.0631); 473.0697 (100%) (calc. for $[Zn(ACA)_2 + H]^+$ = 473.0686); 495.0492 (100%) (calc. for $[Zn(ACA)_2 + Na]^+$ = 495.0505); 740.0591 (79%) (calc. for $[Zn_2(ACA)_3]^+$ = 740.0559); 967.1126 (74%) (calc. for $[Zn_2(ACA)_4 + Na]^+$ = 967.1118); 504.0268 (40%) (calc. for $[Zn_3(ACA)_4]^{2+}$ = 504.0253). FTIR-ATR (wavenumber, $cm^{-1}$): 3581(w) [ν(O-H)], 3496(w) [ν(O-H)], 3212(w) [ν(N-H)], 3154-3007(br) $[\nu(C\text{-}H)]_{ar}$ + $[\nu(C\text{-}H)]_{alk}$, 2944-2802(br) $[\nu(C\text{-}H)]_{al}$, 1632(m) [ν(C=O)], 1613(w), 1585(m) [$\nu_{as}(COO)$], 1523(s) [$\nu_{as}(COO)$], 1493(m) [ν(C=C/C=N)], 1449(w), 1410(s) [$\nu_s(COO)$], 1363(s) [δ(C=C/C=N)], 1339(sh), 1285(s), 1216(m), 1150(w), 1138(w), 1068(w) [$\delta_{ip}(C\text{-}H)$], 1041(w), 1013(w) [$\delta_{ip}(C\text{-}H)$], 985(w) [$\delta_{ip}(C\text{-}H)$], 927(w), 900(w), 850(w), 826(m),

780(m), 767(m), 750(m), 729(w), 692(s) [$\delta_{oop}$(C-H)], 642(m) [$\delta_{oop}$(C-H)], 609(m), 591(w), 570(s), 526(m). $^1$H NMR (300 MHz; DMSO-$d_6$; $Me_4Si$; 298 K): δ = 9.17 [4H, s, N$H_{ACA}$], 8.73 [4H, dd, $^3$J = 4.5 Hz, $^4$J = 1.7 Hz, *o*-$H_{4,4'\text{-bipy}}$], 7.84 [4H, dd, $^3$J = 4.5 Hz, $^4$J = 1.7 Hz, *m*-$H_{4,4'\text{-bipy}}$], 7.50 [8H, d, $^3$J = 7.3 Hz, *o*-$H_{ACA}$], 7.35 [8H, t, $^3$J = 7.4 Hz, *m*-$H_{ACA}$], 7.29 [4H, d, $^3$J = 7.1 Hz, *p*-$H_{ACA}$], 7.24 [2H, s, NH-C-C$H_{ACA}$], 1.95 [6H, s, CO-C$H_{3,ACA}$]. $^{13}$C{$^1$H} NMR (75 MHz; DMSO-$d_6$; $Me_4Si$; 298 K): δ = 170.5 [NH-*C*$O_{ACA}$], 168.4 [*C*$OO_{ACA}$], 150.6 [*o*-$C_{4,4'\text{-bipy}}$], 144.5 [N-CH-CH-$C_{4,4'\text{-bipy}}$], 135.1 [$O_2$C-$C_{ACA}$], 129.7 [HN-C-CH-$C_{ACA}$], 129.3 [*o*-$C_{ACA}$], 129.0 [*p*-$C_{ACA}$], 128.3 [*m*-$C_{ACA}$], 128.2 [NH-C-*C*$H_{ACA}$], 121.4 [*m*-$C_{4,4'\text{-bipy}}$], 23.0 [CO-*C*$H_{3,ACA}$]. DEPT-135 NMR (75 MHz; DMSO-$d_6$; $Me_4Si$, 298 K): δ = 150.6 [*o*-$C_{4,4'\text{-bipy}}$], 129.3 [*o*-$C_{ACA}$], 129.0 [*p*-$C_{ACA}$], 128.3 [*m*-$C_{ACA}$], 128.2 [NH-C-*C*$H_{ACA}$], 121.4 [*m*-$C_{4,4'\text{-bipy}}$], 23.0 [CO-*C*$H_{3,ACA}$].

**{[$Zn_3$(μ-ACA)$_6$(4,4'-bipy)]·0.75$CHCl_3$}$_n$ (4).** 9.7 mg (59.0%) (based on Zn). Elemental analysis calc(%). for $C_{76.75}H_{68.75}Zn_3N_8O_{18}Cl_{2.25}$ (1667.10): C 55.29; H 4.17; N 6.72; found: C 55.63; H 4.21; N 6.79. HR-MS (ESI$^+$, MeOH): m/z (%) = 157.0768 (100%) (calc. for [4,4'-bipy + H]$^+$ = 157.0760); 228.0630 (100%) (calc. for [HACA + Na]$^+$ = 228.0631); 473.0697 (100%) (calc. for [Zn(ACA)$_2$ + H]$^+$ = 473.0686); 495.0492 (100%) (calc. for [Zn(ACA)$_2$ + Na]$^+$ = 495.0505); 740.0591 (79%) (calc. for [$Zn_2$(ACA)$_3$]$^+$ = 740.0559); 967.1126 (74%) (calc. for [$Zn_2$(ACA)$_4$ + Na]$^+$ = 967.1118); 504.0268 (40%) (calc. for [$Zn_3$(ACA)$_4$]$^{2+}$ = 504.0253). FTIR-ATR (wavenumber, cm$^{-1}$): 3242(w) [ν(N-H)], 3163-3025(br) [ν(C-H)$_{ar}$] + [ν(C-H)$_{alk}$], 2956-2853(br) [ν(C-H)$_{al}$], 1677(w), 1660(w), 1646(w) [ν(C=O)], 1589(s), 1573(s) [$\nu_{as}$(COO)], 1532(m) [ν(C=C/C=N)], 1492(w) [ν(C=C/C=N)], 1446(w), 1389(s) [$\nu_s$(COO)], 1365(s) [δ(C=C/C=N)], 1351(sh), 1278(m), 1226(w), 1210(w), 1184(w), 1158(w), 1123(w), 1080(w) [$\delta_{ip}$(C-H)], 1030(sh), 1018(w) [$\delta_{ip}$(C-H)], 980(w) [$\delta_{ip}$(C-H)], 962(w), 927(w), 847(w), 810(w), 790(w), 763(s), 751(m), 731(w), 688(s) [$\delta_{oop}$(C-H)], 673(sh), 643(w) [$\delta_{oop}$(C-H)], 619(w), 604(m), 592(s), 575(s), 524(m). $^1$H NMR (300 MHz; DMSO-$d_6$; $Me_4Si$; 298 K): δ = 9.20 [6H, s, N$H_{ACA}$], 8.73 [4H, dd, $^3$J = 4.5 Hz, $^4$J = 1.7 Hz, *o*-$H_{4,4'\text{-bipy}}$], 7.84 [4H, dd, $^3$J = 4.5 Hz, $^4$J = 1.7 Hz, *m*-$H_{4,4'\text{-bipy}}$], 7.50 [12H, d, $^3$J = 7.1 Hz, *o*-$H_{ACA}$], 7.36 [12H, t, $^3$J = 7.3 Hz, *o*-$H_{ACA}$], 7.29 [2H, d, $^3$J = 6.9 Hz, *m*-$H_{ACA}$], 7.23 [2H, s, NH-C-C$H_{ACA}$], 1.95 [6H, s, CO-C$H_{3,ACA}$]. $^{13}$C{$^1$H} NMR (75 MHz; DMSO-$d_6$; $Me_4Si$; 298 K): δ = 170.5 [NH-*C*$O_{ACA}$], 168.6 [*C*$OO_{ACA}$], 150.7 [*o*-$C_{4,4'\text{-bipy}}$], 144.6 [N-CH-CH-$C_{4,4'\text{-bipy}}$], 135.1 [$O_2$C-$C_{ACA}$], 129.7 [HN-C-CH-$C_{ACA}$], 129.4 [*o*-$C_{ACA}$], 129.1 [*p*-$C_{ACA}$], 128.5 [*m*-$C_{ACA}$], 128.3 [NH-C-*C*$H_{ACA}$], 121.5 [*m*-$C_{4,4'\text{-bipy}}$], 23.1 [CO-*C*$H_{3,ACA}$]. DEPT-

135 NMR (75 MHz; DMSO-$d_6$; $Me_4Si$, 298 K): δ = 150.7 [$o$-$C_{4,4'\text{-bipy}}$], 129.4 [$o$-$C_{ACA}$], 129.2 [$p$-$C_{ACA}$], 128.5 [$m$-$C_{ACA}$], 128.3 [NH-C-$CH_{ACA}$], 121.5 [$m$-$C_{4,4'\text{-bipy}}$], 23.1 [CO-$CH_{3,ACA}$].

**X-ray crystallographic refinement data**

The X-ray intensity data were measured on a D8 Venture system equipped with a multilayer monochromator and a Mo microfocus (λ = 0.71073 Å). For **1C**, the integration of the data using a triclinic unit cell yielded a total of 11749 reflections to a maximum θ angle of 31.38° (0.68 Å resolution), of which 11749 were independent (average redundancy 1.000, completeness = 96.0%, $R_{int}$ = 5.87%, $R_{sig}$ = 0.38%) and 10950 (93.20%) were greater than 2σ($|F|^2$). The calculated minimum and maximum transmission coefficients (based on crystal size) are 0.6903 and 0.7461. For **2**, the integration of the data using a monoclinic unit cell yielded a total of 25688 reflections to a maximum θ angle of 30.61 (0.70 Å resolution), of which 5003 were independent (average redundancy 5.135, completeness = 99.7%, $R_{int}$ = 11.50%, $R_{sig}$ = 9.84%) and 3044 (60.84%) were greater than 2σ($|F|^2$). The calculated minimum and maximum transmission coefficients (based on crystal size) are 0.6646 and 0.7461. For **3**, the integration of the data using a monoclinic unit cell yielded a total of 52017 reflections to a maximum θ angle of 30.59° (0.70 Å resolution), of which 7846 were independent (average redundancy 6.630, completeness = 98.3%, $R_{int}$ = 2.72%, $R_{sig}$ = 1.70%) and 7481 (95.35%) were greater than 2σ($|F|^2$). The calculated minimum and maximum transmission coefficients (based on crystal size) are 0.6688 and 0.7461. For **4C**, the integration of the data using a triclinic unit cell yielded a total of 70704 reflections to a maximum θ angle of 26.49° (0.80 Å resolution), of which 11166 were independent (average redundancy 6.332, completeness = 99.2%, $R_{int}$ = 16.53%, $R_{sig}$ = 10.86%) and 7193 (64.42%) were greater than 2σ($|F|^2$). The calculated minimum and maximum transmission coefficients (based on crystal size) are 0.6774 and 0.7454.

The structures were solved and refined using a SHELXTL Software Package (version-2018/3)[1]. For **1C**, the final anisotropic full-matrix least-squares refinement on $|F|^2$ with 461 variables converged at $R_1$ = 4.03%, for the observed data and $wR_2$ = 9.98% for all data. For **2**, the final anisotropic full-matrix least-squares refinement on $|F|^2$ with 216 variables converged at $R_1$ = 5.70%, for the observed data and $wR_2$ = 13.63% for all data. For **3**, the final anisotropic full-matrix least-squares refinement on $|F|^2$ with 351 variables converged at $R_1$ = 3.47%, for the observed data and $wR_2$ = 9.15% for all data.

For **4C**, the final anisotropic full-matrix least-squares refinement on $|F|^2$ with 630 variables converged at $R_1$ = 7.34%, for the observed data and $wR_2$ = 14.69% for all data.

For **1C**, **2**, **3** and **4C** the final cell constants and volume are based upon refinement of the XYZ-centroids of reflections above 20 σ(I). Data were corrected for absorption effects using the Multi-Scan method (SADABS). Molecular graphics were generated using Mercury 4.3.1 software[2], using the POV-Ray image package[3]. The color codes for all of the molecular graphics are as follows: dark blue (Zn), red (O), light blue (N), light green (Cl), gray (C), and white (H). All the accessible void volumes have been calculated with Mercury 4.3.1 software[2] using a probe radius of 1.2 Å[4]. The evaluation of the geometry distortion of Zn(II) *cores* in **1C**, **2**, **3** and **4C** has been done using version 2.1 of SHAPE software from the corresponding .cif files[5].

**Obtention of single crystals of 1C**

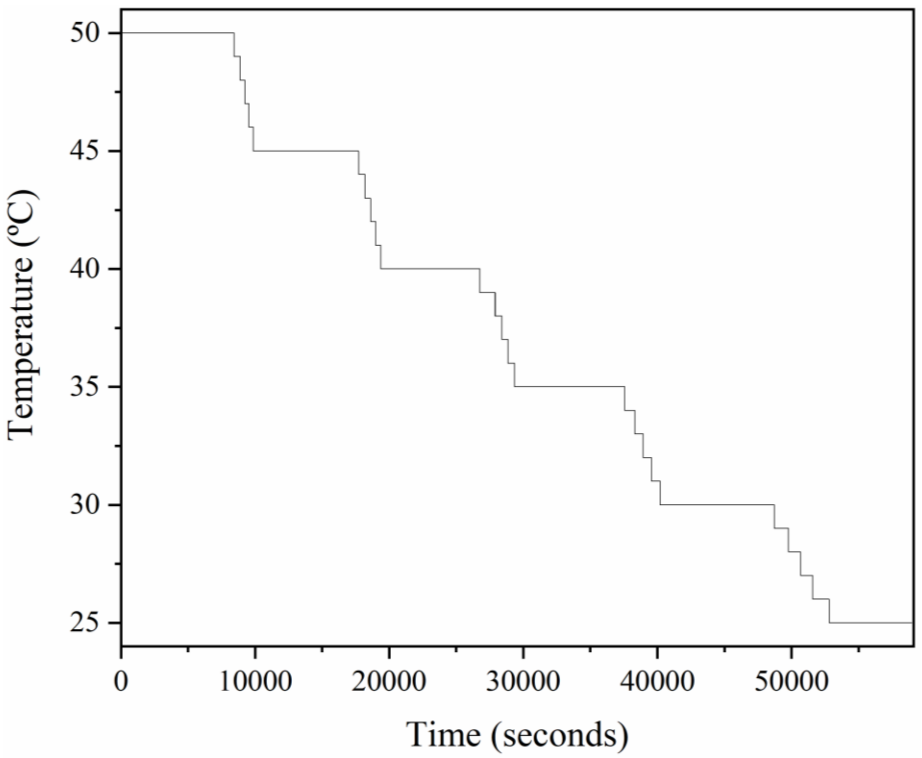


Figure S1. Cooling ramp used for the obtention of single crystals of compound {[Zn(ACA)$_2$(4,4'-bipy)] 2.5EtOH}$_n$ (**1C**).

**PXRD patterns**

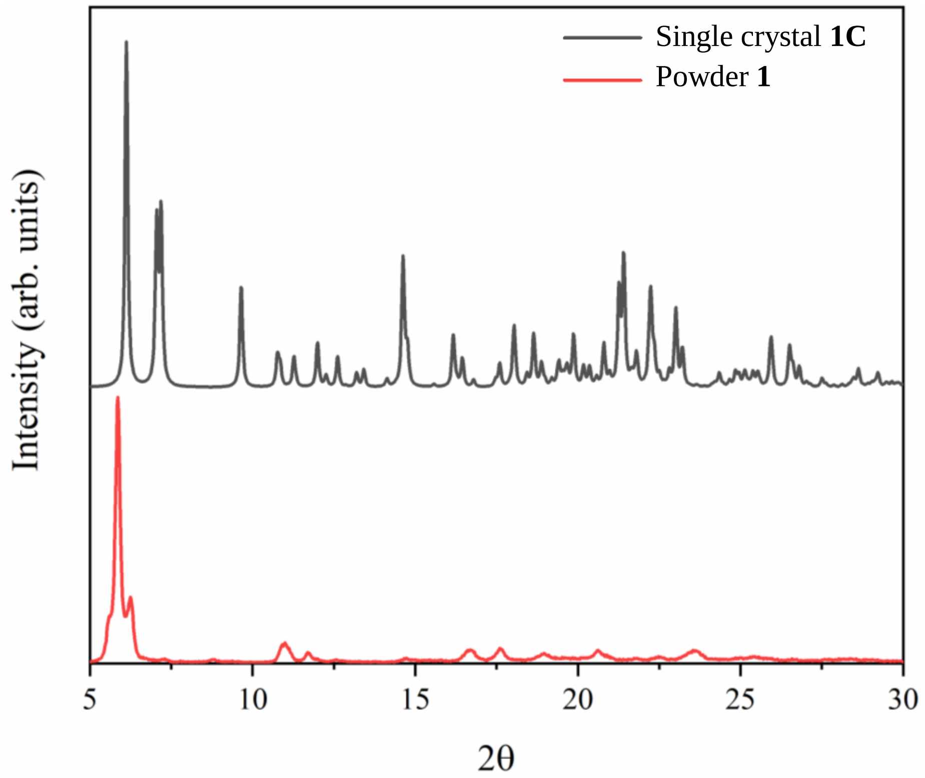


Figure S2. XRD patterns from the single crystal collected data at 100K of {[Zn(ACA)$_2$(4,4'-bipy)]·2.5EtOH}$_n$ (**1C**) and powder XRD pattern at 298K of compound {[Zn(ACA)$_2$(4,4'-bipy)]·EtOH}$_n$ (**1**).

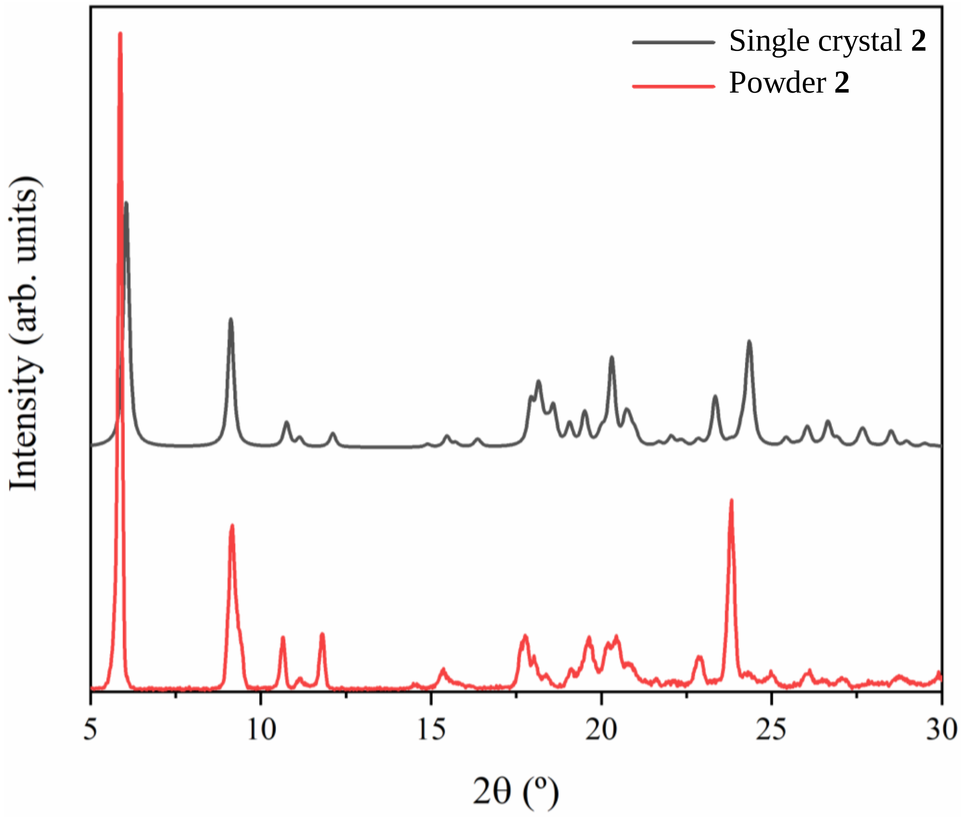


Figure S3. XRD patterns from the single crystal collected data at 100K and powder XRD pattern at 298K of compound {[Zn(ACA)$_2$(4,4'-bipy)]·2MeOH}$_n$ (**2**).

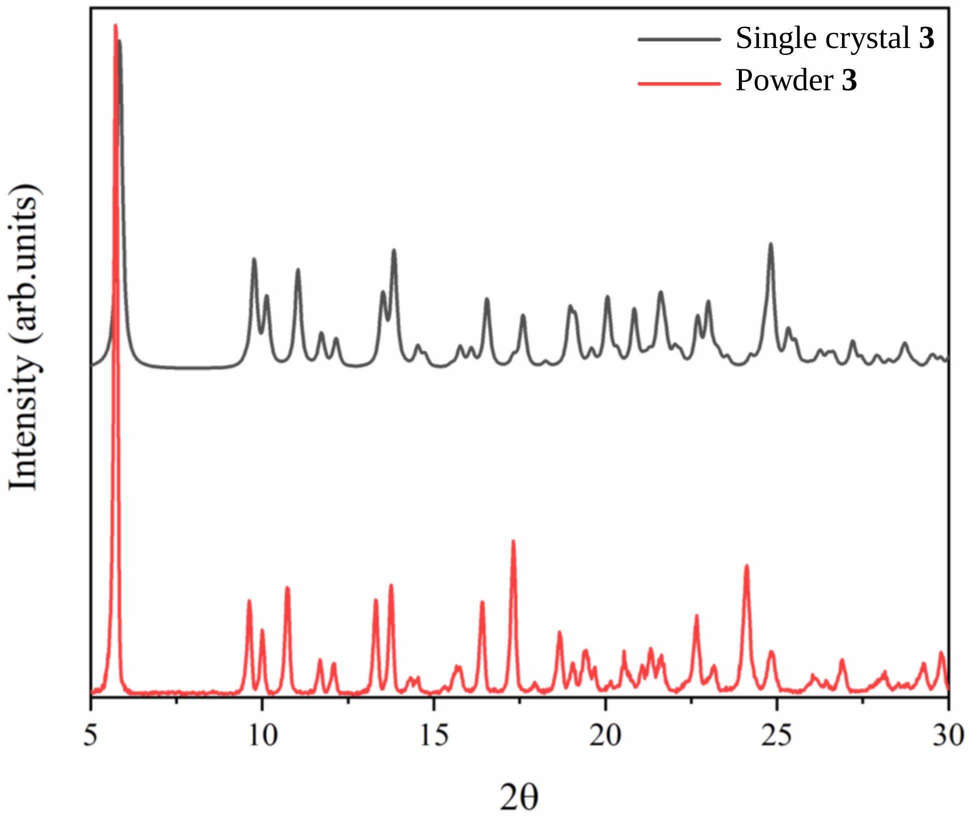


Figure S4. XRD patterns from the single crystal collected data at 100K and powder XRD pattern at 298K of compound {[$Zn_2$(μ-ACA)$_2$(ACA)$_2$(4,4'-bipy)]·$2H_2O$}$_n$ (**3**).

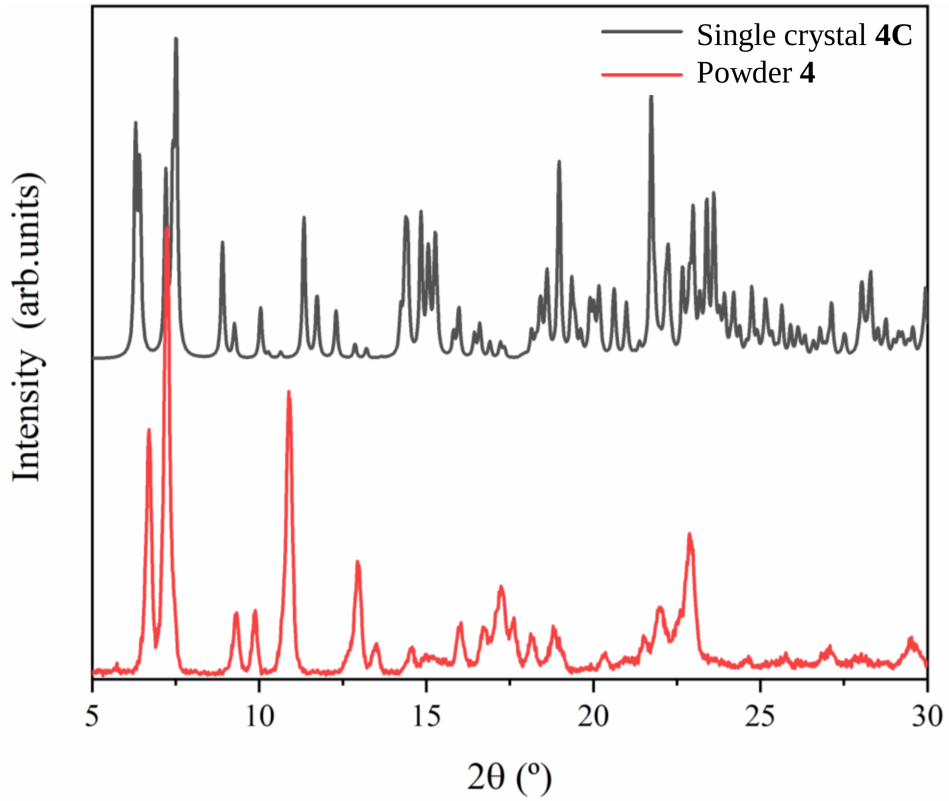


Figure S5. XRD patterns from the single crystal collected data at 100K of {[$Zn_3$(μ-ACA)$_6$(4,4'-bipy)]·$8CHCl_3$}$_n$ (**4C**) and powder XRD pattern at 298K of compound {[$Zn_3$(μ-ACA)$_6$(4,4'-bipy)]·$0.75CHCl_3$}$_n$ (**4**).

## Thermogravimetric analysis

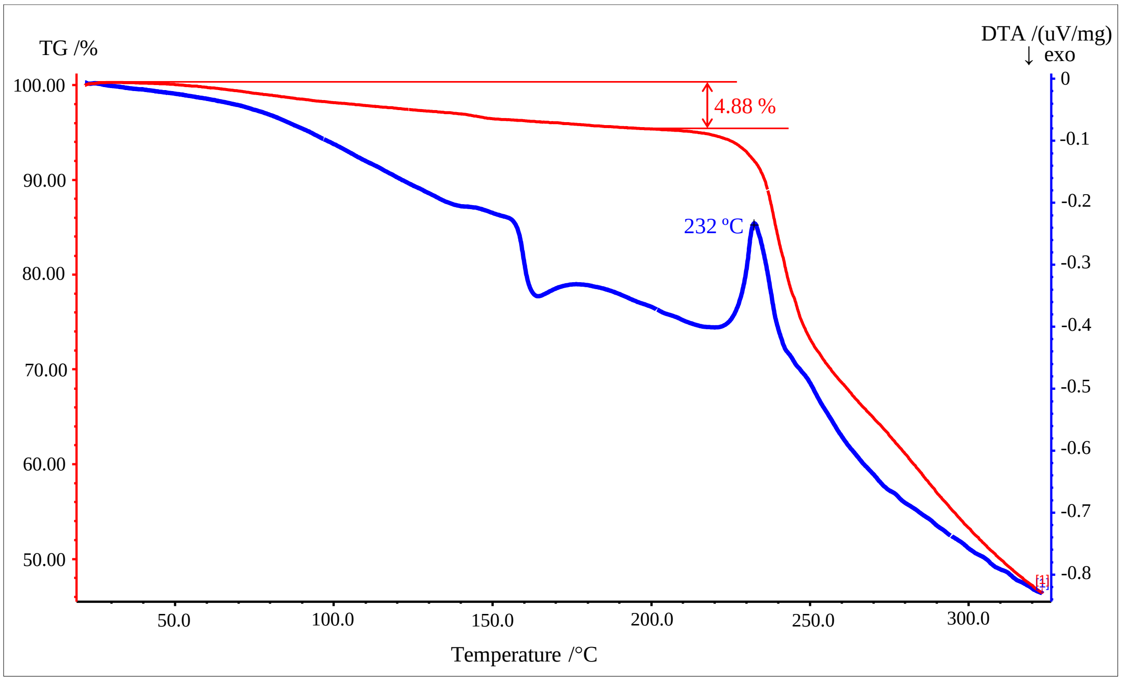


Figure S6. TG-DTA of compound {[Zn(ACA)$_2$(4,4'-bipy)]·EtOH}$_n$ (**1**).

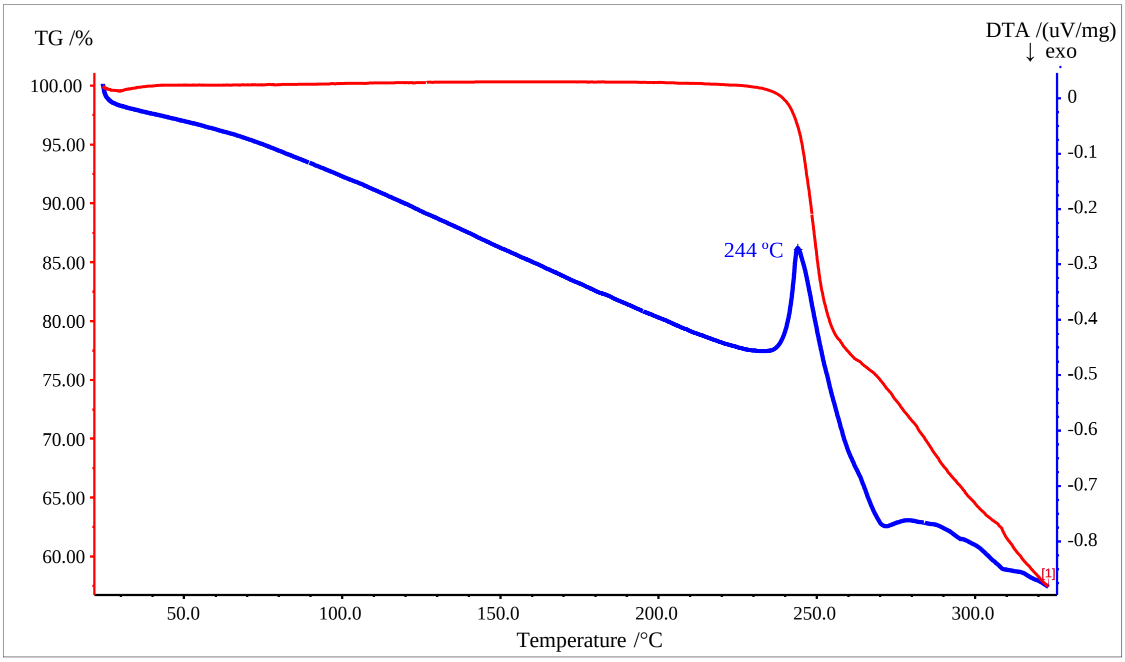


Figure S7. TG-DTA of compound {[Zn$_3$(μ-ACA)$_6$(4,4'-bipy)]·0.75CHCl$_3$}$_n$ (**4**).

# HR-ESI-MS

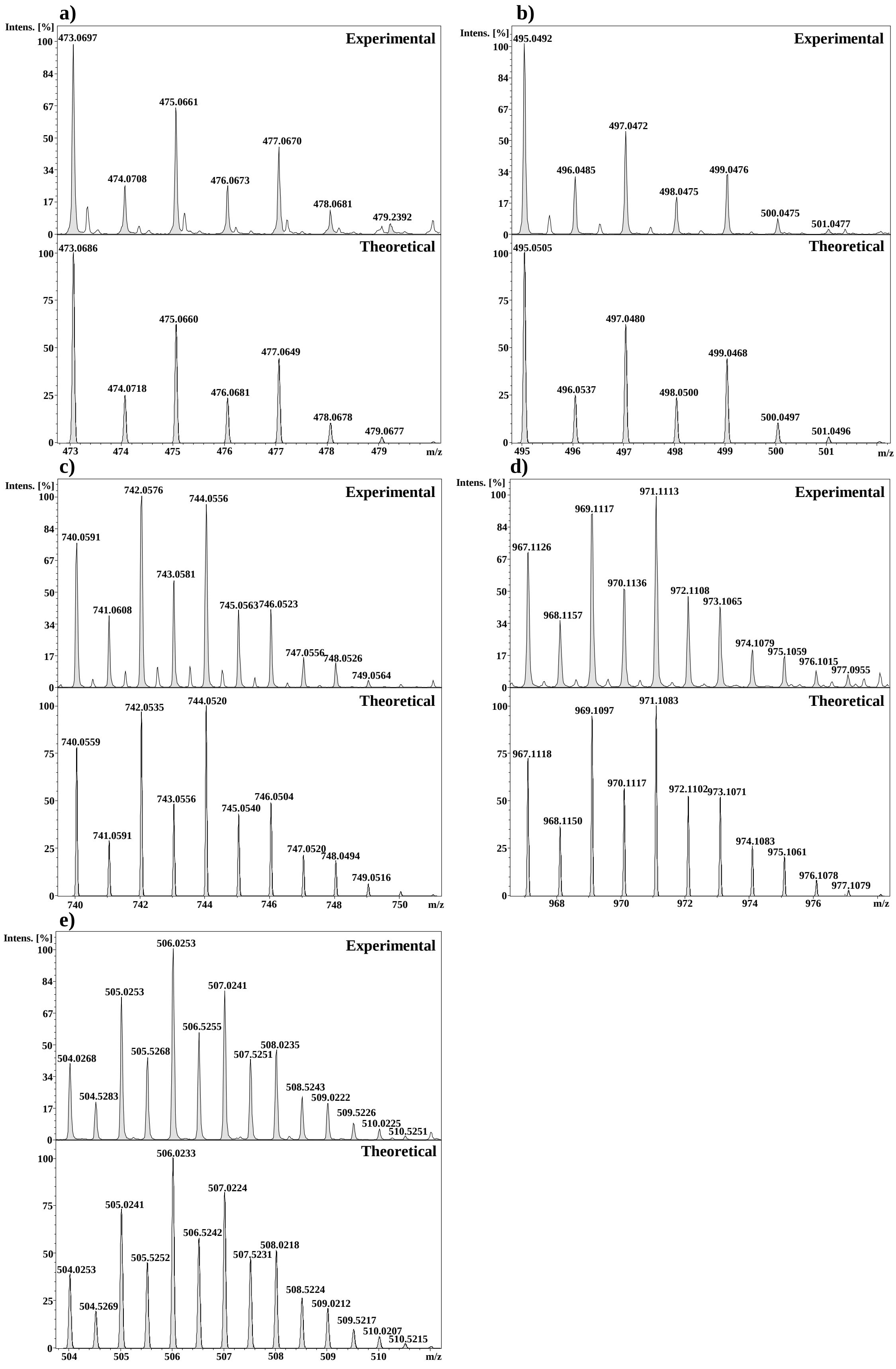


Figure S8. In detail views of the HR-ESI-MS fragments of **1**-**4**: (a) $[Zn(ACA)_2+H]^+$. (b) $[Zn(ACA)_2+Na]^+$. (c) $[Zn_2(ACA)_3]^+$. (d) $[Zn_2(ACA)_4 + Na]^+$. (e) $[Zn_3(ACA)_4]^{2+}$.

**FTIR-ATR, ¹H, ¹³C{¹H} and DEPT-135 NMR spectroscopies**

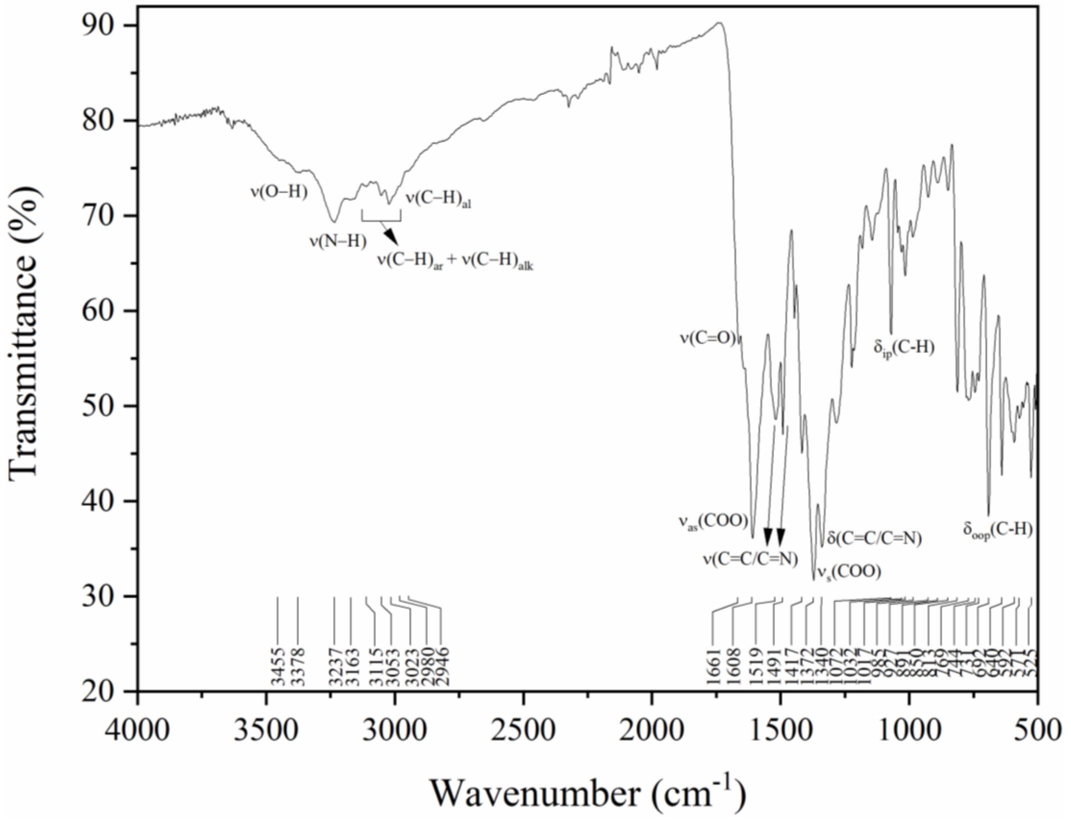

Figure S9. FTIR-ATR spectrum of compound {[Zn(ACA)$_2$(4,4'-bipy)]·EtOH}$_n$ (**1**).

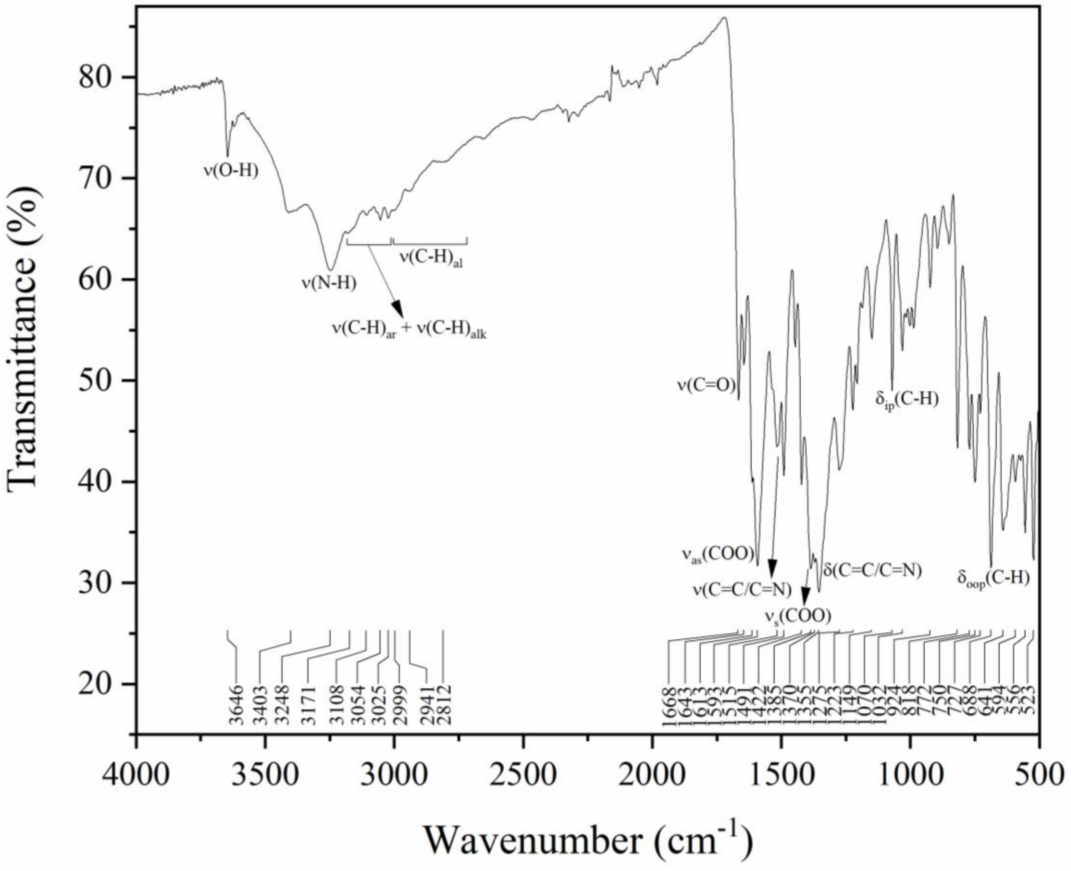

Figure S10. FTIR-ATR spectrum of compound {[Zn(ACA)$_2$(4,4'-bipy)]·2MeOH}$_n$ (**2**).

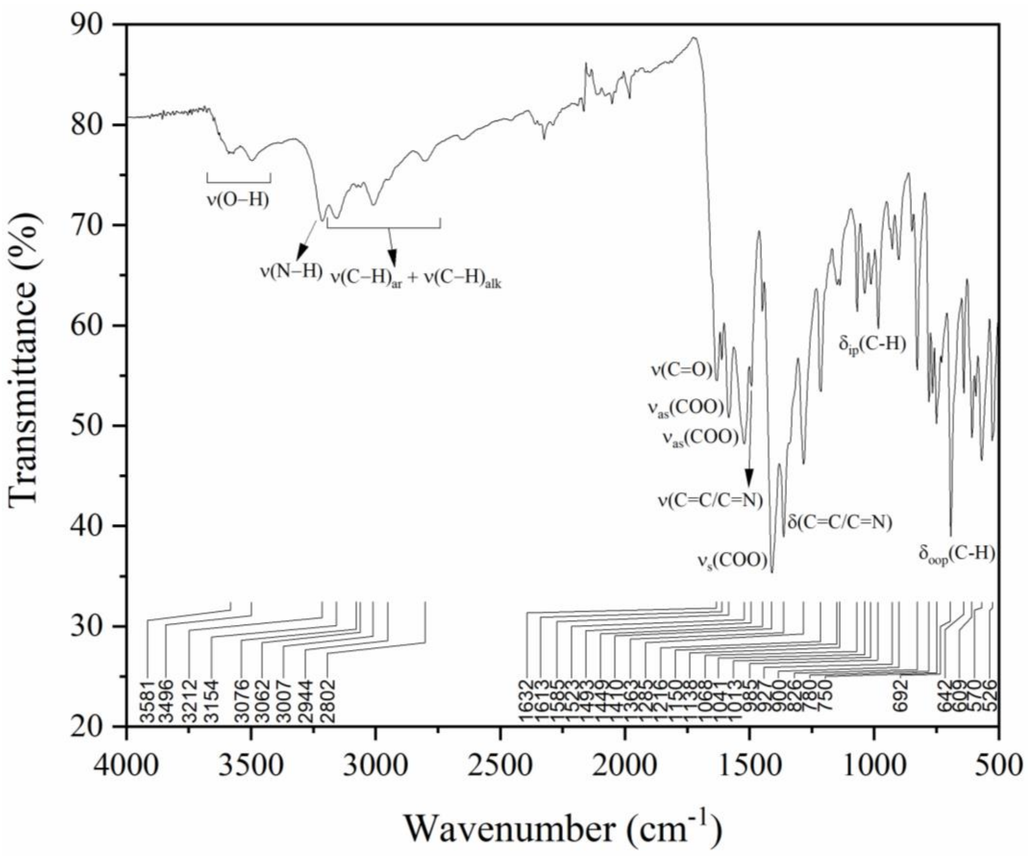


Figure S11. FTIR-ATR spectrum of compound $\{[Zn_2(\mu\text{-}ACA)_2(ACA)_2(4,4'\text{-}bipy)]\cdot 2H_2O\}_n$ (**3**).

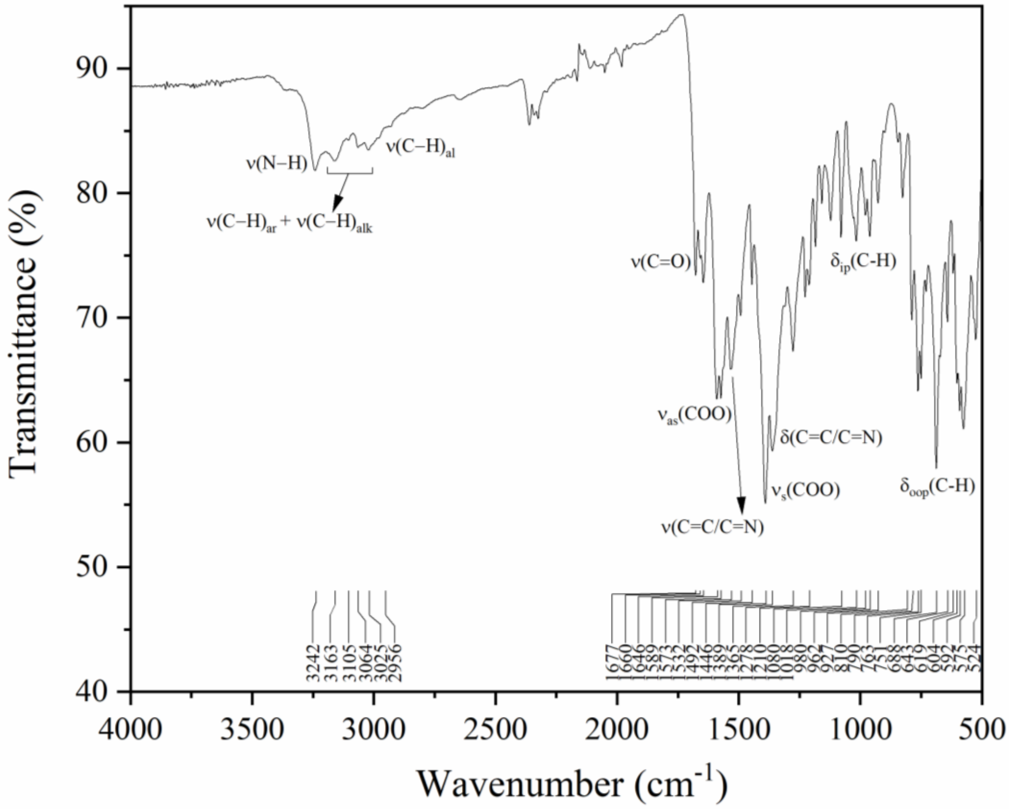


Figure S12. FTIR-ATR spectrum of compound $\{[Zn_3(\mu\text{-}ACA)_6(4,4'\text{-}bipy)]\cdot 0.75CHCl_3\}_n$ (**4**).

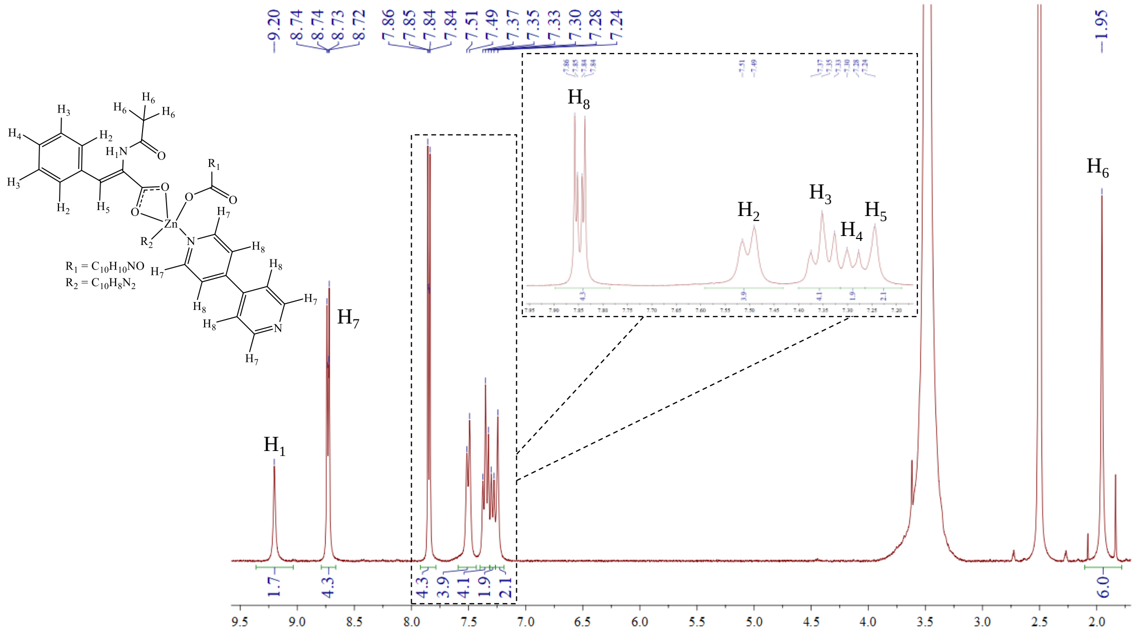

Figure S13. $^{1}H$ NMR spectrum of compound $\{[Zn(ACA)_2(4,4'\text{-bipy})]\cdot EtOH\}_n$ (**1**).

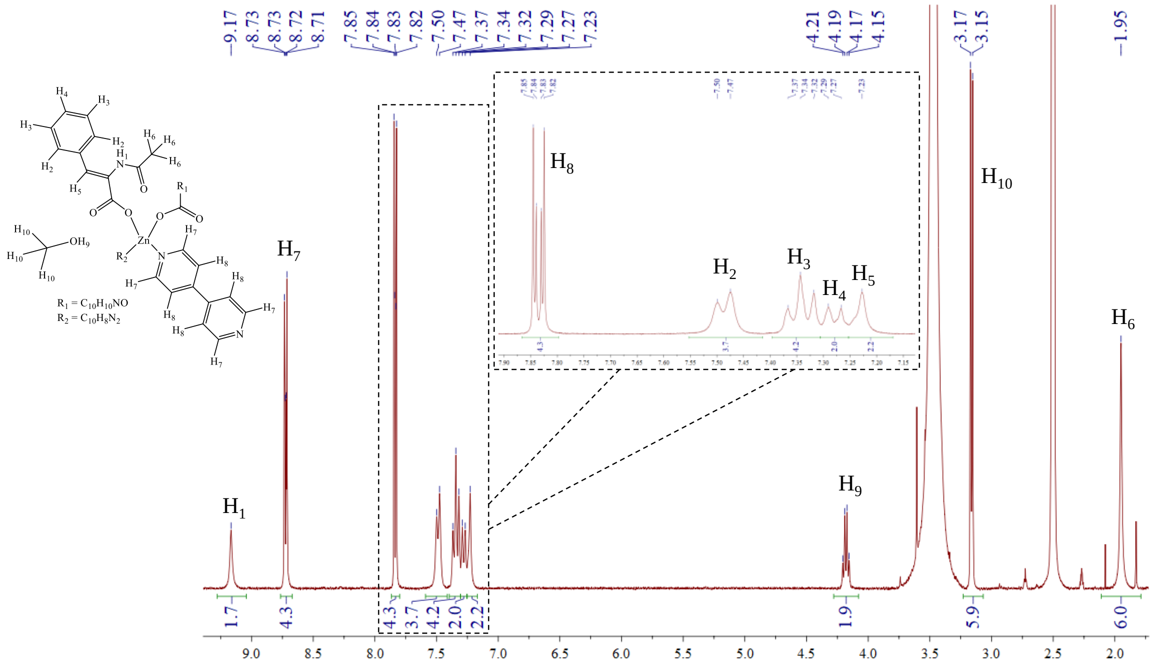

Figure S14. $^{1}H$ NMR spectrum of compound $\{[Zn(ACA)_2(4,4'\text{-bipy})]\cdot 2MeOH\}_n$ (**2**).

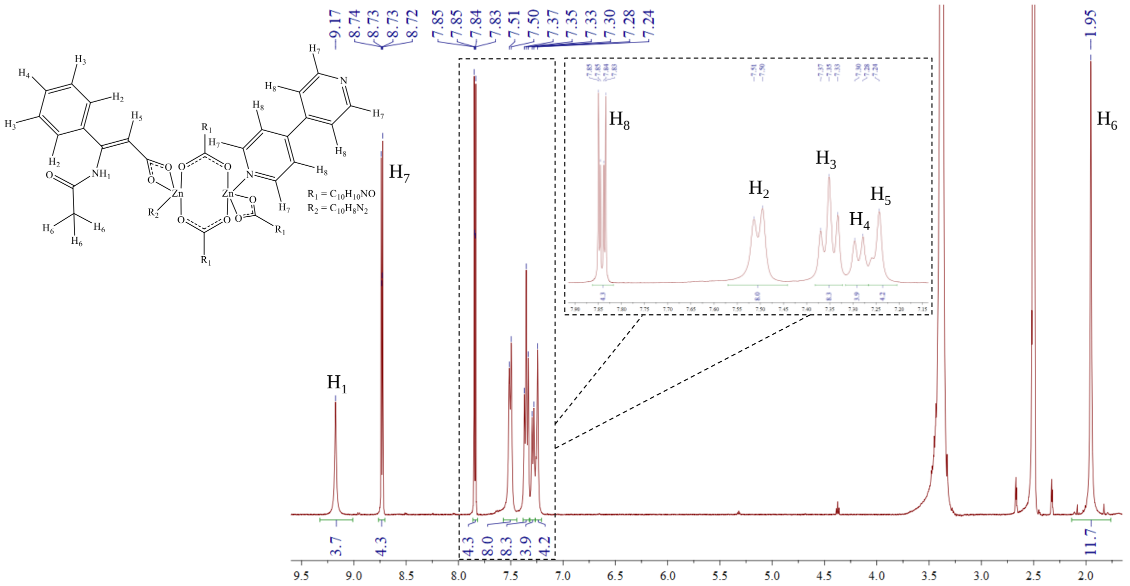


Figure S15. $^{1}$H NMR spectrum of compound $\{[Zn_2(\mu\text{-}ACA)_2(ACA)_2(4,4'\text{-bipy})]\cdot 2H_2O\}_n$ (**3**).

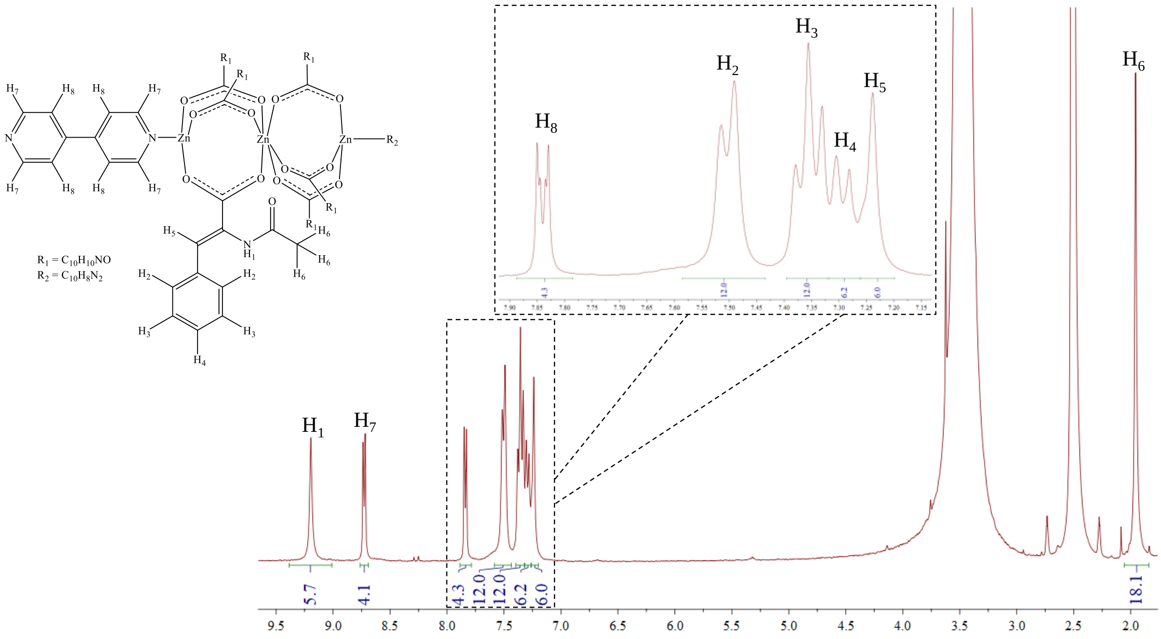


Figure S16. $^{1}$H NMR spectrum of compound $\{[Zn_3(\mu\text{-}ACA)_6(4,4'\text{-bipy})]\cdot 0.75CHCl_3\}_n$ (**4**).

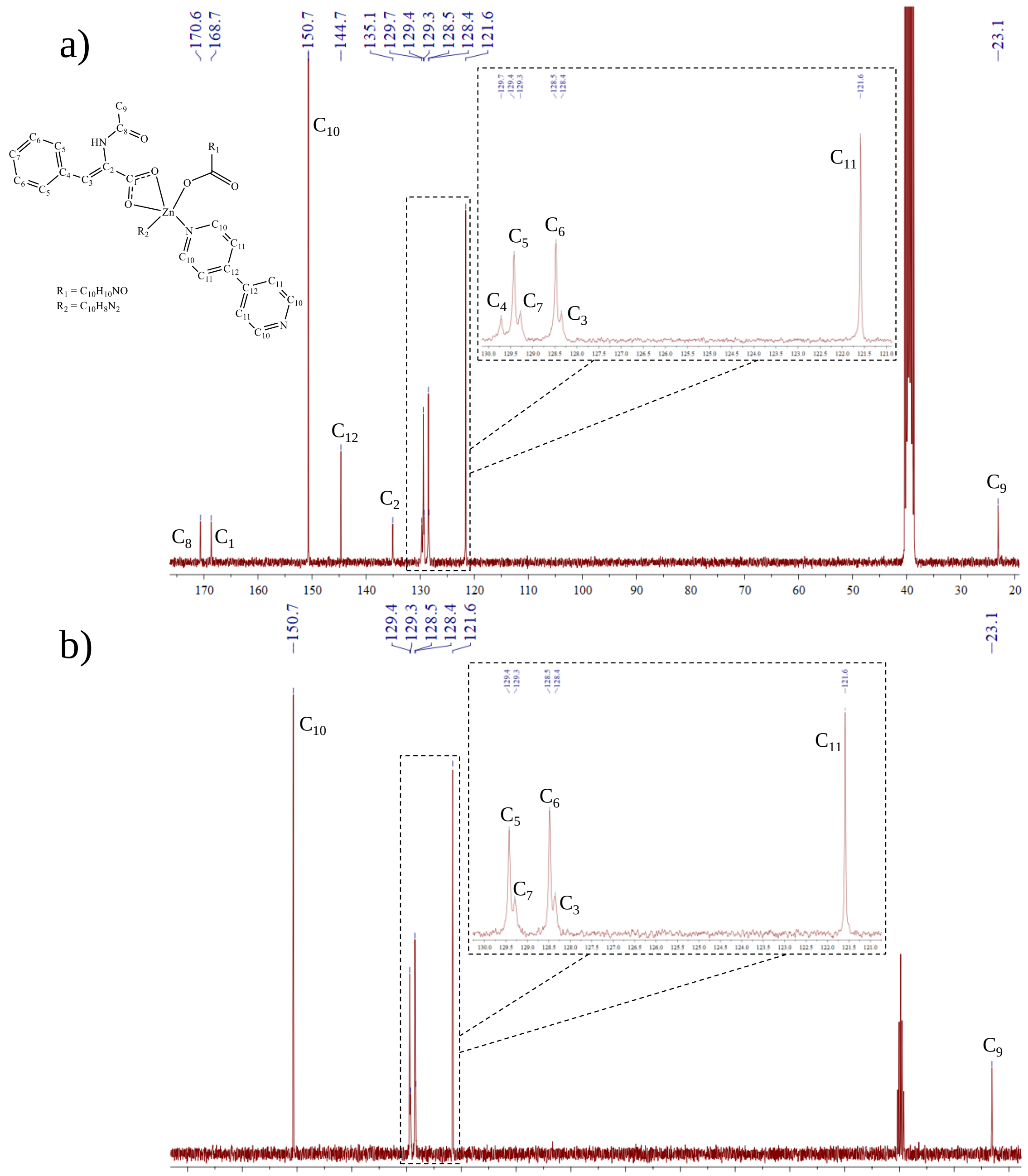


Figure S17. (a) $^{13}C\{^{1}H\}$ and (b) DEPT-135 NMR spectra of compound $\{[Zn(ACA)_2(4,4'\text{-}bipy)]\cdot EtOH\}_n$ (**1**).

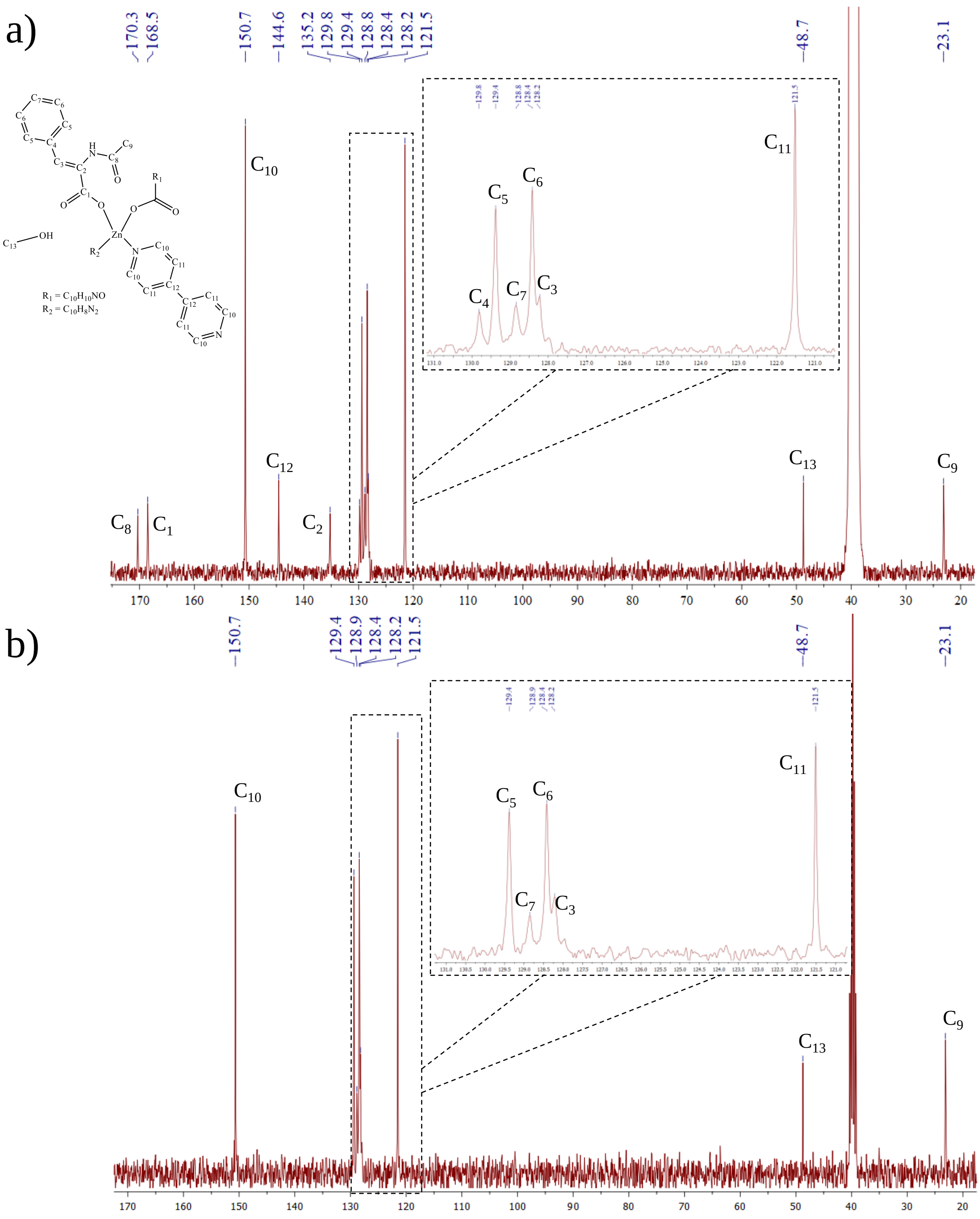


Figure S18. (a) $^{13}C\{^{1}H\}$ and (b) DEPT-135 NMR spectra of compound {[Zn(ACA)$_2$(4,4'-bipy)]·2MeOH}$_n$ (**2**).

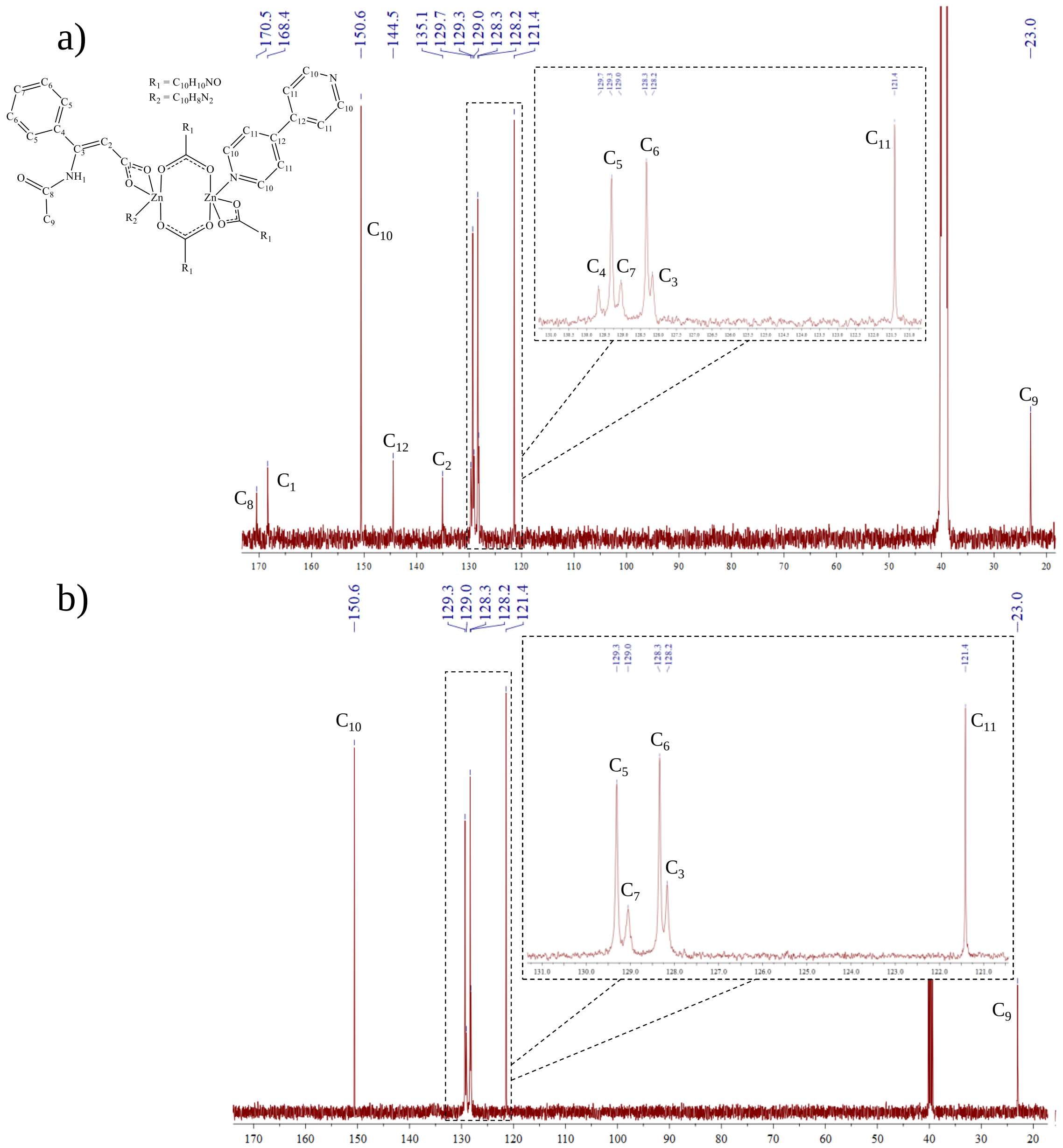


Figure S19. (a) $^{13}C\{^1H\}$ and (b) DEPT-135 NMR spectra of compound $\{[Zn_2(\mu\text{-}ACA)_2(ACA)_2(4,4'\text{-bipy})]\cdot 2H_2O\}_n$ (**3**).

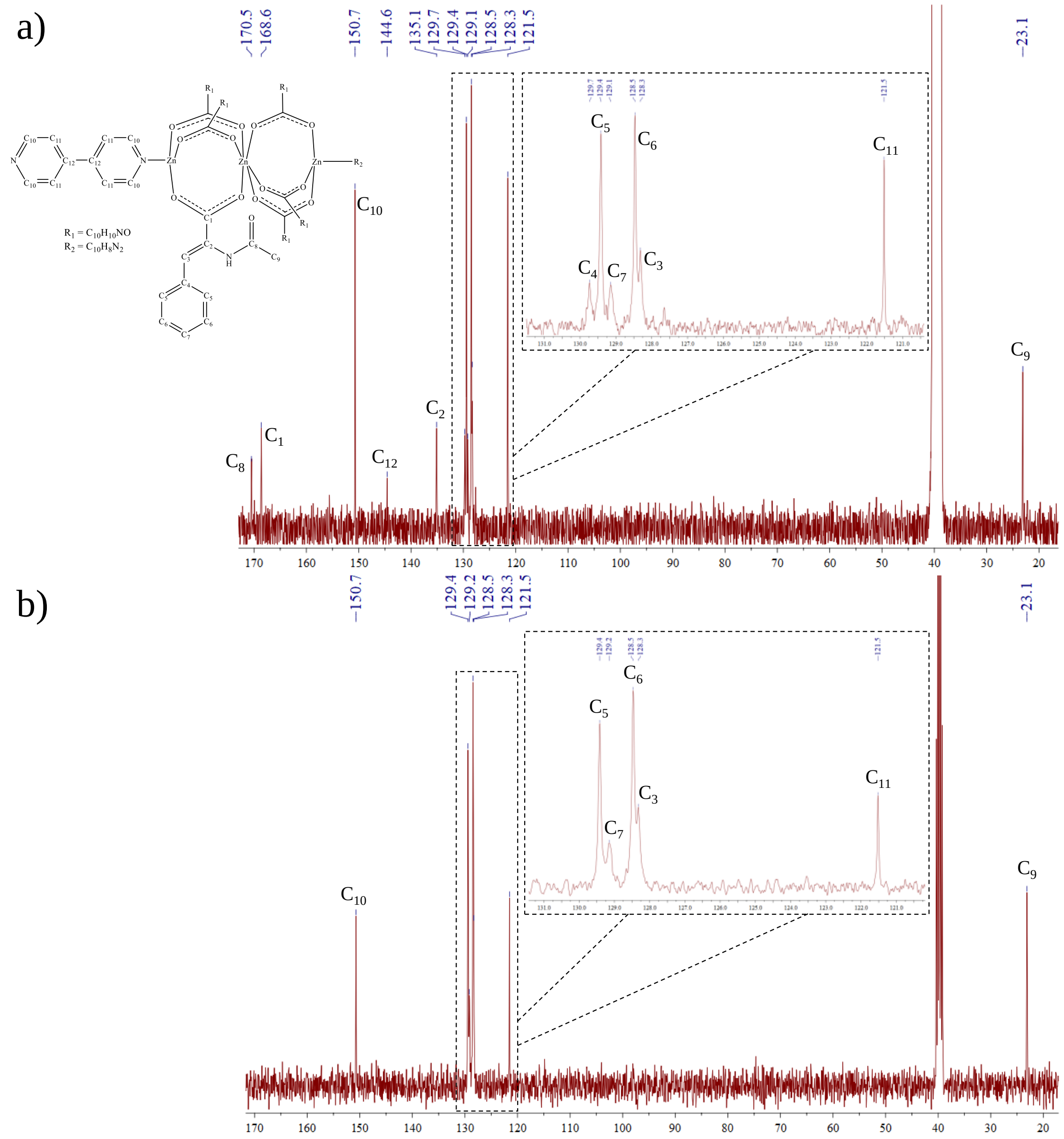


Figure S20. (a) $^{13}C\{^{1}H\}$ and (b) DEPT-135 NMR spectra of compound $\{[Zn_3(\mu\text{-}ACA)_6(4,4'\text{-bipy})]\cdot 0.75CHCl_3\}_n$ (**4**).

## Geometric Evaluation

Table S1. Geometry distortion analysis of the Zn(II) *cores* from **1C**, **2**, **3** and **4C** using *S* parameter calculated with SHAPE[5,6].

| Compound | Label | Geometry[a] | *S* value |
|---|---|---|---|
| **1C** | Zn(1) | **T-4** | **0.946** |
| | | SS-4 | 5.501 |
| | | vTBPY-4 | 1.808 |
| **2** | Zn(1) | **T-4** | **0.630** |
| | | SS-4 | 8.302 |
| | | vTBPY-4 | 2.962 |
| **3** | Zn(1) | PP-5 | 30.204 |
| | | vOC-5 | 4.508 |
| | | TBPY-5 | 4.308 |
| | | **SPY-5** | **2.525** |
| | | JTBPY-5 | 6.230 |
| **4C** | Zn(1) | **T-4** | **0.843** |
| | | SS-4 | 6.764 |
| | | vTBPY-4 | 1.265 |
| | Zn(2) | **OC-6** | **0.245** |
| | | TPR-6 | 16.152 |

Closer values have been highlighted in bold. [a] T-4 = Tetrahedron; SS-4 = Seesaw or sawhorse; vTBPY-4 = Axially vacant trigonal bipyramid; PP-5 = Pentagon; vOC-5 = Vacant octahedron; TBPY-5 = Trigonal bipyramid; SPY-5 = Square pyramid; JTBPY-5 = Johnson trigonal bipyramid; OC-6 = Octahedron; TPR-6 = Trigonal prism.

## Photoluminescence data

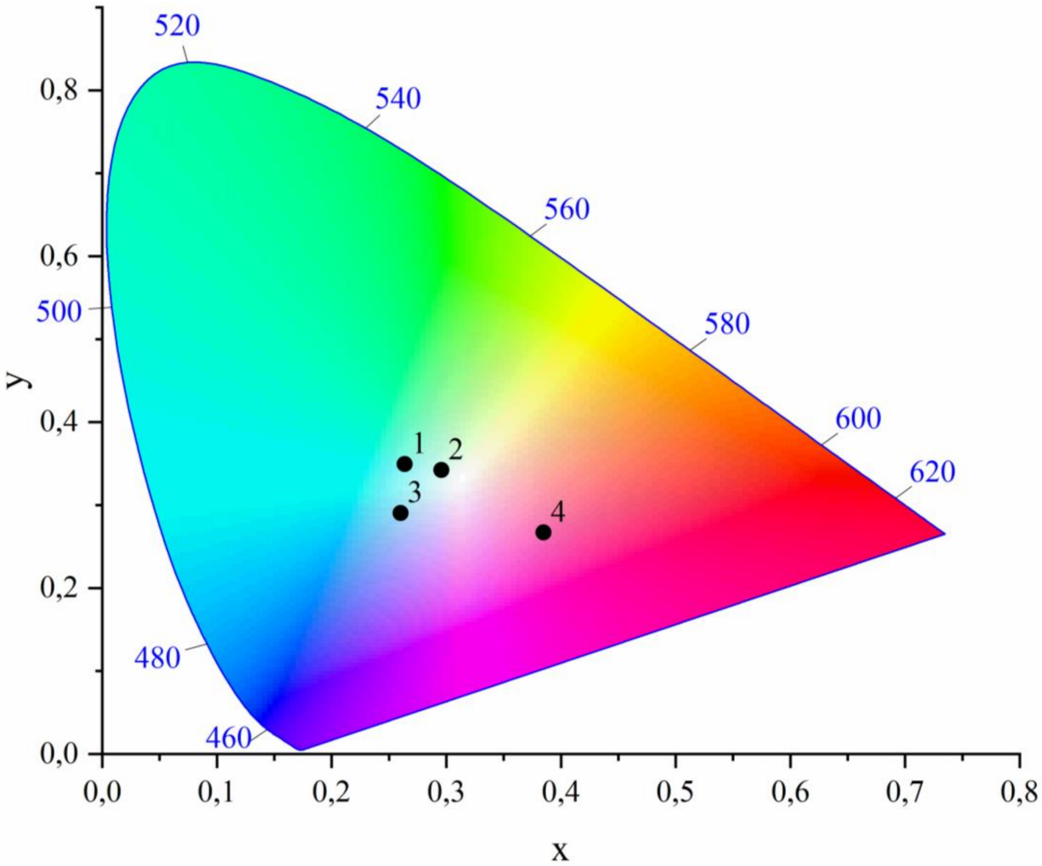


Figure S21. CIE 1931 chromaticity diagram for **1-4**.

## References


(1) Sheldrick, G. M. A Short History of SHELX. *Acta Cryst. Sect. A* **2008**, *64*, 112–122. https://doi.org/10.1107/S0108767307043930.

(2) MacRae, C. F.; Sovago, I.; Cottrell, S. J.; Galek, P. T. A.; McCabe, P.; Pidcock, E.; Platings, M.; Shields, G. P.; Stevens, J. S.; Towler, M.; Wood, P. A. Mercury 4.0: From Visualization to Analysis, Design and Prediction. *J. Appl. Cryst.* **2020**, *53*, 226–235. https://doi.org/10.1107/S1600576719014092.

(3) Persistence of Vision Pty. Ltd. Persistence of Vision (TM) Raytracer; Persistence of Vision Pty. Ltd.: Williamstown, Australia, 2004.

(4) Spek, A. L. Single-Crystal Structure Validation with the Program PLATON. *J. Appl. Cryst.* **2003**, *36*, 7–13. https://doi.org/10.1107/S0021889802022112.

(5) Llunell, M.; Casanova, D.; Cirera, J.; Alemany, P.; Alvarez, S. *SHAPE. Program for the Stereochemical Analysis of Molecular Fragments by Means of Continuous Shape Measures and Associated Tools*. Universitat de Barcelona, Barcelona 2013.

(6) Pinsky, M.; Avnir, D. Continuous Symmetry Measures. 5. The Classical Polyhedra. *Inorg. Chem.* **1998**, *37*, 5575–5582. https://doi.org/10.1021/ic9804925.
